# Ruxolitinib-based senomorphic therapy mitigates cardiomyocyte senescence in septic cardiomyopathy by inhibiting the JAK2/STAT3 signaling pathway

**DOI:** 10.7150/ijbs.96489

**Published:** 2024-08-12

**Authors:** Boshen Yang, Taixi Li, Zhixiang Wang, Yuankang Zhu, Kaifan Niu, Sien Hu, Zhiqi Lin, Xinjie Zheng, Xian Jin, Chengxing Shen

**Affiliations:** 1 Department of Cardiology, Shanghai Sixth People's Hospital Affiliated to Shanghai Jiao Tong University School of Medicine, Shanghai, China.; 2 Institute for Developmental and Regenerative Cardiovascular Medicine, Xinhua Hospital, School of Medicine, Shanghai Jiao Tong University, Shanghai, China.; 3 Department of Respiratory Medicine, The Fourth Affiliated Hospital, Zhejiang University School of Medicine, Yiwu, China.; 4 Department of Surgery, The Fourth Affiliated Hospital, Zhejiang University School of Medicine, Yiwu, Zhejiang, China.

**Keywords:** Cellular senescence, septic cardiomyopathy, senomorphic therapy, mitochondrial dysfunction, oxidative stress.

## Abstract

**Background:** Cellular senescence has emerged as a pivotal focus in cardiovascular research. This study investigates the previously unrecognized role of cellular senescence in septic cardiomyopathy (SCM) and evaluates senomorphic therapy using ruxolitinib (Rux) as a potential treatment option.

**Methods:** We employed lipopolysaccharide (LPS)-induced neonatal rat cardiomyocytes (NRCMs) and two mouse models—LPS-induced and cecal ligation and puncture (CLP)-induced SCM models—to assess Rux's effects. RNA sequencing, western blotting (WB), quantitative polymerase chain reaction (qPCR), immunofluorescence, immunohistochemistry, senescence-associated β-galactosidase (SA-β-gal) assay, and other techniques were utilized to investigate underlying mechanisms.

**Results:** Senescence-associated secretory phenotype (SASP) and cellular senescence markers were markedly elevated in LPS-induced NRCMs and SCM animal models, confirmed by the SA-β-gal assay. Rux treatment attenuated SASP *in vitro* and *in vivo*, alongside downregulation of senescence markers. Moreover, Rux-based senomorphic therapy mitigated mitochondrial-mediated apoptosis, improved cardiac function in SCM mice, restored the balance of antioxidant system, and reduced reactive oxygen species (ROS) levels. Rux treatment restored mitochondrial membrane potential, mitigated mitochondrial morphological damage, and upregulated mitochondrial complex-related gene expression, thereby enhancing mitochondrial function. Additionally, Rux treatment ameliorated SCM-induced mitochondrial dynamic dysfunction and endoplasmic reticulum stress. Mechanistically, Rux inhibited JAK2-STAT3 signaling activation both *in vitro* and *in vivo*. Notably, low-dose Rux and ABT263 showed comparable efficacy in mitigating SCM.

**Conclusions:** This study highlighted the potential significance of cellular senescence in SCM pathogenesis and suggested Rux-based senomorphic therapy as a promising therapeutic approach for SCM.

## Introduction

Cellular senescence is a condition of permanent cessation of cell division linked to the process of aging^1^. Age-related telomere shortening is a significant factor in replicative senescence, but senescence can also be triggered by oxidative stress, metabolic dysfunction, and epigenetic regulation, among other factors^1^. Recently, stress-induced cellular senescence has been discovered to play a role in various cardiovascular disorders such as myocardial infarction (MI), myocardial ischemia-reperfusion (IR) damage, and heart failure^2^, ^3^. Senescent cardiomyocytes have reduced contractile ability, elevated pacing frequency, impaired shortening, and the capacity to release senescence-associated secretory phenotype (SASP) factors that can induce senescence in neighboring cells^1^. Notably, researchers have discovered that utilizing Navitoclax to pharmacologically eliminate senescent cells in mice after MI reduced age-related myocardial remodeling and decreased the production of profibrotic mediators^4^. Similarly, targeting senescence has been shown to be an effective and practical approach to reduce maladaptive remodeling and enhance recovery after IR injury^5^. These findings indicated that stress-induced cellular senescence might be a significant factor in cardiovascular disease. However, it is still unclear whether cellular senescence plays a role in the context of septic cardiomyopathy (SCM).

As a serious consequence of sepsis, SCM affects 18%-65% of cases and has a high mortality rate of 36%-55%^6^, which is characterized by impaired left ventricular (LV) contractility that ultimately leads to LV dilatation with or without right ventricular failure^7^. It's interesting to note that a major characteristic of aging cardiomyocytes is a reduction in contractile capacity, which also occurs in SCM^8^, ^9^. Although cardiomyocyte senescence has not been studied in the context of SCM, multiple-organ senescence induced by sepsis has been widely reported. For instance, Chen *et al.* discovered that premature senescence was a major factor in sepsis-induced acute kidney injury (AKI), and that in a mouse model, reducing the interaction between inflammation and premature senescence had renoprotective benefits^10^. Li *et al.* found that augmented cellular senescence was observed in lung tissue and contributed to higher mortality in mice following cecal ligation and puncture (CLP)^11^. Cellular senescence was also demonstrated to be associated with sepsis-induced muscle weakness^12^. In light of these findings, we hypothesize that cellular senescence, particularly cardiomyocyte senescence, plays a significant role in SCM.

Oxidative stress and mitochondrial dysfunction are two fundamental mechanisms shared by both cellular senescence and SCM. Excessive production of mitochondrial reactive oxygen species (mtROS) during sepsis further impeded mitochondrial activity and led to structural rupture 13. Furthermore, elevated ROS levels caused biomacromolecular damage to DNA, lipids, proteins, and other molecules, which accelerated cellular senescence^14^. Moreover, the generation of ROS could also initiate endoplasmic reticulum (ER) stress, subsequently promoting cellular senescence^15^. Mitochondrial dynamics disorders occurred in septic hearts, and maintaining their balance has been proven to be a beneficial strategy for SCM^16^. In human bronchial epithelial cells, disruption of mitochondrial dynamics through knockdown of fusion proteins, such as OPA1 and MFNs, led to mitochondrial fragmentation, elevated ROS levels, and promoted cellular senescence induced by smoke extract^17^. Muscle-specific deletion of Opa1 induced a precocious senescence phenotype and premature death in mouse models^18^. Furthermore, persistent accumulation of Drp1 in cardiomyocytes reduced ATP production and increased ROS generation, ultimately leading to myocardial senescence and chronic heart failure^19^. These studies indicated that targeting oxidative stress and restoring mitochondrial dynamics could be a common strategy for alleviating SCM and reducing cellular senescence.

Ruxolitinib (Rux) is a typical senomorphic agent that has been proven to be effective in alleviating cellular SASP without killing senescent cells, distinguishing it from senolytic drugs^20^, ^21^. In a mouse model of chronic obstructive pulmonary disease, treatment with Rux caused a reversal of cellular senescence, lung emphysema, and fibrosis^22^. Administration of Rux to aged mice alleviated both adipose tissue and systemic inflammation and enhanced physical function^21^. By blocking SASP, Rux increased bone regeneration in elderly rats and brought it back to levels akin to those of young rats^23^. Rux is also a well-known JAK inhibitor, and JAK-STAT pathway is considered to play a crucial role in cellular senescence. Researchers discovered that in late cartilage-derived stem/progenitor cells, pharmacological suppression or genetic knockdown of the JAK-STAT signaling pathway reduced cellular senescence^24^. Additionally, the senescence of bone marrow-mesenchymal stem cells associated with systemic lupus erythematosus might be significantly influenced by the JAK2-STAT3 signaling pathway^25^. Herein, we assume that Rux can alleviate cardiomyocyte senescence by inhibiting SASP of cardiomyocytes, thereby alleviating sepsis-induced cardiac injury.

Our goal in this work is to investigate cellular senescence in relation to SCM. In particular, it is important to determine whether cellular senescence, especially in cardiomyocytes, occurs in SCM. If so, does Rux-based senomorphic treatment reduce myocardial senescence induced by sepsis, thereby alleviating septic heart dysfunction. Our research may offer a new perspective on treating SCM by examining the previously unrecognized role of cellular senescence in this condition.

## Materials and Methods

### Regents

The Rux powder (A3012, APEXBIO, USA), a monomer with a purity of 99.79%, was bought from APEXBIO Co., Ltd. The highest solubility of the Rux powder in dimethyl sulfoxide (DMSO) was 15.32 mg/mL. We dissolved the Rux powder in DMSO (D8371-50, Solarbio, China) to create the primary solution. The primary solution was diluted with Dulbecco's Modified Eagle's Medium (DMEM) for *in vitro* experiments or corn oil for *in vivo* investigations. The Lipopolysaccharides (LPS, L2880, Sigma, USA) were bought from Sigma-Aldrich Co., Ltd and dissolved in saline before being employed in experiments. Recombinant IL-6 (rIL-6) was purchased from MedChemExpress Co., Ltd (HY-P7103A, MCE, China). Navitoclax (ABT-263) was bought from InvivoChem Co., Ltd (923564-51-6, InvivoChem, USA). These regents were stored and prepared according to the manufacturer's instructions.

### Cell culture and treatment

Neonatal rat cardiomyocytes (NRCMs) were extracted from the ventricles of 1-to-3-day-old neonatal Sprague-Dawley rats obtained from Slac Laboratory Animal Co., Ltd. (Shanghai, China) following a previously outlined method (23). Simply put, the neonatal rats were given anesthesia, and their hearts were perfused with ice-cold phosphate-buffered saline (PBS) to eliminate blood cells. The hearts were then dissected, minced, and digested with collagenase II (Gibco, USA) at 37°C. Following centrifugation, resuspension, and differential adherent isolation, bromodeoxyuridine (BrdU, Beyotime, China) was introduced to purify cardiomyocytes. The NRCMs were grown in DMEM supplemented with 10% fetal bovine serum (FBS) and 1% penicillin-streptomycin solution.

10 μg/mL LPS was utilized to stimulate NRCMs for 12 hours to establish an *in vitro* model of SCM. We separated the *in vitro* experiment into four groups to explore the therapeutic effect of Rux on LPS-induced NRCMs. It has been reported that the Rux corresponding to the maximum human plasma concentration is adjusted to 1.51 μM, and CCK8 experiments have shown that it did not exhibit cytotoxicity to NRCMs until the Rux concentration reaches 100 μM 26. Therefore, we chose 1 μM as the drug concentration for *in vitro* experiments. The study included a control group of NRCMs, a group of NRCMs treated with 1μM of Rux, a group of LPS-induced NRCMs, and a group of LPS-induced NRCMs treated with 1μM of Rux. The Rux therapy began 2 hours after LPS stimulation. In order to reactivate the JAK2/STAT3 pathway, 20 ng/mL of r-IL6 was used and added simultaneously with Rux 2 hours after LPS stimulation^27^. Equal volume of DMSO or PBS were added to each group to prevent the influence of confounding variables. D-galactose (D-gal) and hydrogen peroxide (H_2_O_2_) were employed in our cell experiments to further validate the therapeutic efficacy of Rux across different cellular models. Detailed protocols are provided in the Supplementary Materials for reference.

### Animal models and experimental procedures

We purchased 6- to 8-week-old male C57BL/6 mice from SLAC ANIMAL (Shanghai, China). This study conducted two types of animal models to mimic SCM: LPS-induced and CLP-induced mouse models. An intraperitoneal injection of a single dose of 10 mg/kg LPS was administered to elicit SCM in mouse models. We meticulously weighed each mouse before injection to ensure consistent levels of LPS administration. The surgery for CLP-induced mouse models was conducted as outlined in a previous study^28^. Briefly, 75% medicinal alcohol was used to clean the mouse abdomen before conducting CLP surgery under isoflurane with oxygen. The polymicrobial sepsis model involved partially ligating the cecum of the individuals and puncturing it with a 22-gauge needle. After puncturing, an equivalent volume of feces was expelled, and then the cecum was returned to the abdominal cavity. After the wound was patched, the mice were injected with 1 mL of sterile saline immediately following the procedure. For CLP models, our technicians possessed extensive experience and maintained blinding to the experimental groups. The control group underwent the identical surgical procedure without ligation and puncture. No antibiotics were administered to any of the experimental mice. 2 hours and 6 hours after LPS injection or CLP surgery, mice in the treatment groups were administered 30 mg/kg or 75 mg/kg of Rux via intragastric administration. The control group received an equal amount of saline. The dosage of Rux chosen for *in vivo* experiments is determined based on previous research^26^. The procedure for animal experiments regarding ABT263 was consistent with a previous study^29^.

### Western blotting

To initiate the lysis process, the cells or heart tissue were subjected to RIPA buffer (G2002, Servicebio, China). The protein concentration of the cell lysate or cardiac tissue in this investigation was measured to be 2 μg/μl. The typical quantity of total cell or tissue protein was 20 μg. Subsequently, the extracted proteins underwent SDS-PAGE and were then transferred onto a PVDF membrane (WGPVDF45, Servicebio, China). Following this, the samples were incubated overnight at a temperature of 4°C with the appropriate primary antibodies. Afterward, the samples were incubated with secondary antibodies conjugated with horseradish peroxidase (33101es60, Yeasen, China) at a temperature of 25°C for 1 hour. The signals were evaluated using the enhanced chemiluminescence (ECL) system (180-506, Tanon, China). The protein signal was quantified using the Image J software. The loading control chosen for this study was glyceraldehyde-3-phosphate dehydrogenase (GAPDH). **Table S1** provides a summary of the primary antibodies employed in this study.

### Senescence associated β-galactosidase (SA-β-gal) staining

SA-β-gal activity was measured in accordance with manufacturer's instructions using Senescence associated β-Galactosidase Staining Kit (#9860, Cell Signaling Technology, USA). To put it briefly, mouse hearts were divided into 10 μm frozen tissue sections and embedded in optimal cutting temperature compound. After fixing the sections for 15 minutes at room temperature using a fixative solution, they were incubated with a β-gal staining solution overnight at 37°C, with a final pH of 6.0. The staining of NRCMs follows a similar procedure. Cells and stained slices were examined using a Keyence VHX-7000 microscope (Keyence, Japan).

### Real-time PCR

The Trizol-based approach was employed to extract total RNA from heart tissue or cells. Subsequently, the collected RNA was transformed into complementary DNA (cDNA) using HiScript II Q RT SuperMix for quantitative polymerase chain reaction (qPCR) with a gDNA wiper (R223-01, Vazyme, China). Quantitative RT-PCR studies were performed using the lightCycler® 96 Instrument (Roche, Switzerland) and the ChamQTM SYBR Color qPCR Master Mix (Q411-02, Vazyme, China). Every result was normalized using the hypoxanthine-guanine phosphoribosyl transferase (HPRT) assay. **Table S2** provides a summary of the primer sequences employed in the present study.

### Immunohistochemistry and immunofluorescence

The immunohistochemical procedure encompassed several steps, including deparaffinization, antigen retrieval, peroxidase blocking, circling, serum blocking, overnight primary antibody incubation at 4°C, and subsequent secondary antibody incubation at room temperature. Microscopic examinations were ultimately conducted to evaluate the stained tissue slices.

The heart tissue chips undergo dewaxing and rehydration for immunofluorescence examination. A solution of citric acid sodium buffer was introduced to facilitate the recovery of antigens. Cultured cells underwent a washing process using phosphate buffered saline (PBS) followed by fixation using a 4% paraformaldehyde solution. Subsequently, the heart tissue chips or cells that had undergone washing were subjected to permeabilization with 0.1% Triton X-100 and subsequently blocked with 3% bovine serum. The samples were stained using one or more antibodies that were specific to the samples. The confocal microscope (Zeiss, German) was utilized to conduct fluorescence measurements. Quantitative analysis was conducted by applying the Image J software to five randomly selected fields of vision from each cardiac slice. An investigator who was unaware of all the groups conducted the measurements.

### Procedures of RNA sequencing (RNA-seq) and data analysis

Eight heart tissues were collected from two groups of mice: the SCM group (undergoing CLP surgery and treated with saline) and the Rux group (undergoing CLP surgery and treated with 75 mg/kg of Rux). The RNA-seq procedures were conducted at Beijing Novogene Technology Co., Ltd, which involved sample quality control, library preparation for transcriptome sequencing, and clustering and sequencing. Differentially expressed genes (DEGs) were identified using the DESeq2 R package (version 1.20.0) with an adjusted p-value below 0.05.

The clusterProfiler R package was used to conduct Gene Ontology (GO) enrichment analysis on DEGs, with correction for gene length bias. GO keywords with adjusted p-values below 0.05 were considered significantly enriched by DEGs. KEGG is a database that offers information on the functions and utilities of biological systems at different levels, including molecular data acquired from genome sequencing and other advanced experimental technologies. We used the clusterProfiler R package to assess the statistical enrichment of DEGs in KEGG pathways.

Gene Set Enrichment Analysis (GSEA) is a computational approach used to determine whether a predefined gene set may suggest a significant and consistent difference between two biological states. The genes were ranked according to the degree of differential expression in the two samples, and then the predefined gene sets were tested to determine if they were enriched at the top or bottom of the list. GSEA can capture subtle expression changes. We use the local version of the GSEA analysis tool http://www.broadinstitute.org/gsea/index.jsp.

### TUNEL assay

The samples of heart tissue were sliced into sections that were 8 micrometers thick, put in paraffin, had the wax removed, and then were hydrated once more. After 10 minutes of treatment with 20 μg/ml of proteinase K, the samples were stained using the cell death test kit (12156792910, Roche, Swiss) according to the instructions provided. The tagged cells were imaged in five randomly selected viewing fields using a confocal microscope (AX10, Zeiss, Germany). By dividing the total number of DAPI-stained cells by the number of TUNEL-positive nuclei with concomitant labeling, the proportion of apoptotic cardiomyocytes was ascertained. Using Image J, the measurements were carried out by an investigator who was blind to all groups.

### Echocardiography

Previous studies have shown that in LPS-induced mouse models, the most significant decrease in heart function was observed at 12 hours after LPS injection^30^, while in the CLP model, the most significant decrease was observed at 24 hours post CLP surgery^31^. Hence, we conducted transthoracic echocardiography on mice using an ultrasound machine (Vivid 7, GE, USA) to assess their heart function at these two time points. The mice were induced into a mild anesthetic condition and maintained a heart rate of 450 ± 50 bpm with a concentration of 0.5%-2% isoflurane. The mice were positioned in a supine posture on a heated platform. The measurements were conducted by an observer who was unaware of the groups. Measurements were obtained at the mid-papillary level to calculate various cardiac parameters, including fractional shortening (FS%), ejection fraction (EF%), left ventricular internal dimension in systole (LVIDs), left ventricular internal dimension at end-diastole (LVIDd), left ventricular end-diastolic volume (LVEDV), and left ventricular end-systolic volume (LVESV). LVEDV and LVESV were determined using the biplane area-length approach. The ejection fraction (EF%) was determined using the following formula: EF% = [(LVEDV-LVESV)/LVEDV] × 100%. The 2D-guided left ventricular M mode tracings were taken at the papillary muscle level from the long-axis perspective. These tracings were used to assess the LVIDs and the LVIDd. The formula used to compute left ventricular fractional shortening (FS%) is as follows: The formula to calculate FS% is as follows: FS% = [(LVIDd-LVIDs)/LVIDd] × 100%. At least five photos per mouse heart were examined. Exemplary photographs for each category were exhibited.

### Transmission electron microscopy (TEM)

Initially, newly harvested hearts extracted from mice were subjected to perfusion with a fixing solution for electron microscopy (G1102, Servicebio, China). Subsequently, a solution containing 0.8% potassium ferrocyanide and 2% osmium tetroxide in a 0.1 mol/L sodium cacodylate buffer was utilized for pre-fixation for a duration of 2 hours. Following this, the specimen was washed three times using a sodium cacodylate buffer. Dehydration was performed on the samples by employing a density gradient including alcohol and acetone. Ultimately, the samples were sliced into ultrathin sections measuring 60-80 nm, then stained and left to dry at room temperature for the duration of the night. The morphology of mitochondria in cardiac cells was obtained by imaging and analyzing the sections using a High Contrast Transmission Electron Microscope (Hitachi HT7800, Japan).

### Detection of ROS levels

The commercial kit DCFH-DA (S0033S, Beyotime, China) was used to quantify the ROS levels inside cells. NRCMs were grown on 24-well plates, and the cells were exposed to the appropriate therapies when each well's cell density reached 50%. Subsequently, the cells were treated with 10 μM DCFH-DA and incubated for 30 minutes at 37°C in the dark. After repeatedly rinsing the cells in PBS, the cells were examined under a fluorescent microscope (TH4-200, OLYMPUS, Tokyo, Japan). According to the manufacturer's recommendations, the MitoSox Red Mitochondrial Superoxide Indicator (RM02822, Abclonal, China) was utilized for MitoSox staining in this investigation. Using Image J software, the relative ROS intensity and mean ROS intensity for each group were determined.

### Superoxide dismutase (SOD) activity and malondialdehyde (MDA) levels

Commercial kits (S0101S, S0131S, Beyotime, China) were used to detect the levels of antioxidant enzyme and oxidative stress-related indicator based on the protocol, including the levels of MDA and the activity of SOD. The procedures of assays were in line with the instructions provided by the manufacturers.

### Mitochondrial respiratory chain (MRC) complex Ⅰ activity

The activity of MRC complex Ⅰ was detected using Mitochondrial complex I/NADH-CoQ reductase Activity Assay Kit (Solarbio, BC0515, China). In briefly, approximately 0.1g of heart tissue from each mouse was collected and prepared as test sample, and the changes in absorbance were subsequently detected at a wavelength of 340 nm according to the instructions. The final enzyme activity is calculated based on the protein concentration of the sample, in units of U/mg prot.

### *In vivo* adenoviral gene transfection into myocardium

To establish an animal model overexpressing P16 in the heart, we employed cardiac adenoviral gene transfer. The method for *in vivo* adenoviral delivery followed established protocols^32^. Briefly, a 20 μl solution of adenovirus (Adv) containing 1 × 10^9^ plaque-forming units (pfu) of either adv-GFP or adv-P16 was administered from the apex of the left ventricle to the aortic root. A tourniquet was applied around the aorta and pulmonary artery distal to the catheter tip, and the solution was injected under these conditions. The tourniquet was maintained for 10 seconds to ensure isovolumetric conditions as the heart pumped against a closed system before being released. Hearts were transduced with adenovirus and later subjected to LPS infection after one week.

### Cell viability assay

We conducted a cell viability assay to assess the potential toxicity of the Rux concentrations used in this study on NRCMs as previously described^33^.

### Statistical analysis

Three separate cell tests were carried out to guarantee the validity of the findings in this work. The data's normality was checked using the Shapiro-Wilk test. For statistical analyses, one-way analysis of variance (ANOVA) corrected by Bonferroni post hoc test and Tukey's multiple comparison test were used. P-values less than 0.05 were considered statistically significant. The information was presented as mean ± standard deviation (SD). For all statistical analyses, GraphPad Prism (version 9.5.1) was utilized.

## Results

### Cellular senescence is induced in SCM: evidence from human, animal, and cell models

Initially, we examined human cardiac samples obtained from 11 donors whose hearts did not fail and 20 patients with SCM, using the GSE79962 dataset. As displayed in **Figure S1A-B**, the markers P53 and P21, which are associated with senescence, exhibited a significant increase in septic hearts compared to nonfailing hearts. Additionally, the mRNA levels of genes related to SASP, such as IL-1R1, IL-6R, IL-18R1, TNFα-IP1, CCL2, EDN1, TGFβ1, CXCL8, and MMP-1, were higher in the hearts of patients with SCM compared to nonfailing donors (**Figure S1C**). Furthermore, the KEGG analysis revealed that cellular senescence and the JAK-STAT signaling pathway were identified as biological processes implicated in the pathogenesis of SCM, based on DEGs observed between the two groups (**Figure S1D**). As shown in **Figure S1E-F**, the GESA analysis provided additional evidence that the JAK-STAT signaling pathway and cellular senescence were greatly activated in the hearts of patients with SCM. Taken together, from a human perspective, these results suggested that cellular senescence might play a significant role in the pathophysiology of SCM.

**Figure 1:**
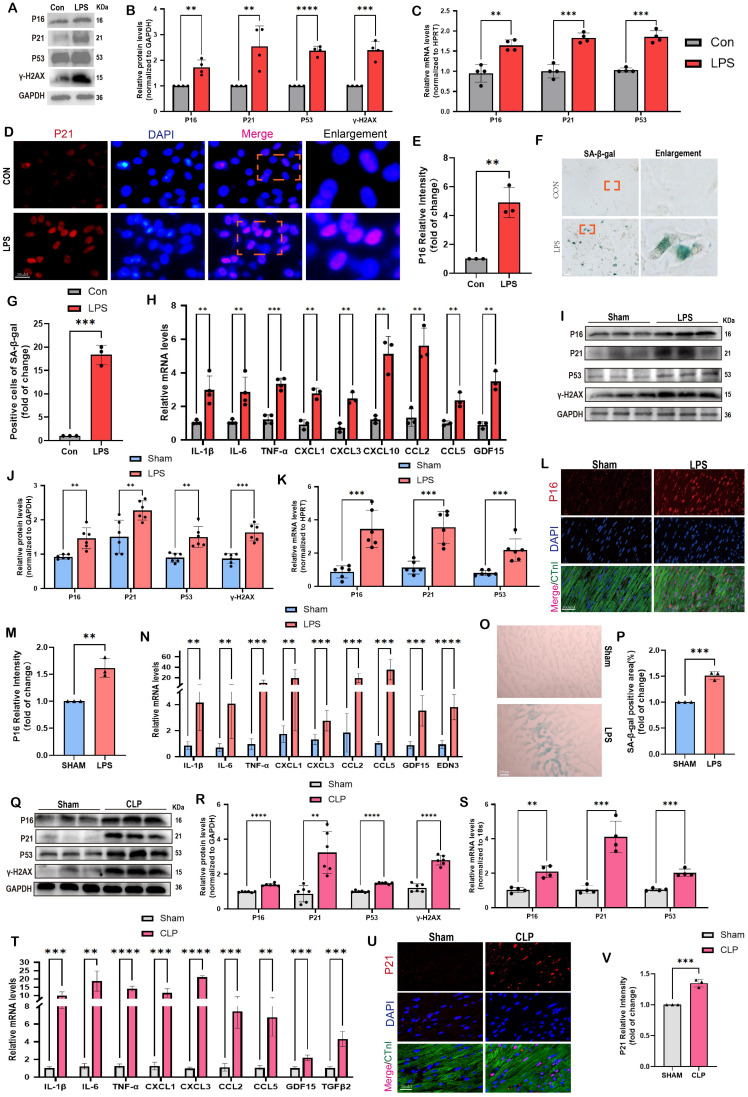
** Cellular senescence is induced in SCM: evidence from human, animal, and cell models.** (A,B) Representative Western blot bands and quantitative analysis of P16, P21, P53, and γ-H2AX in NRCMs between the control and LPS-stimulation groups. N = 4. (C) The mRNA levels of P16, P21 and P53 in NRCMs between the control and LPS-stimulation groups were detected using qRT-PCR. N = 4. (D,E) Representative fluorescence images and quantitative analysis of P16 with DAPI in NRCMs between the control and LPS-stimulation groups. Scale bar = 20 μm, N = 3. (F,G) Representative images and quantitative analysis of SA-β-gal staining in NRCMs. Scale bar = 20 μm, N = 3. (H) The mRNA levels of SASP-related genes were detected using qRT-PCR post LPS-stimulation in NRCMs compared to the control group, including IL-1β, IL-6, TNF-α, CXCL1, CXCL3, CXCL10, CCL2, CCL5 and GDF15. N = 3 - 4. (I,J) Representative Western blot bands and quantitative analysis of P16, P21, P53 and γ-H2AX in LPS-induced mice models compared with the Sham group. N = 6. (K) The mRNA levels of P16, P21, P53 in LPS-induced mice models compared with the Sham group were detected using qRT-PCR. N = 6. (L,M) Representative fluorescence images and quantitative analysis of P16 with cTnI and DAPI in LPS-induced mice models compared with the Sham group. Scale bar = 100 μm, N=3. (N) The mRNA levels of SASP-related genes were detected using qRT-PCR in LPS-induced mice models compared with the Sham group, including IL-1β, IL-6, TNF-α, CXCL1, CXCL3, CCL2, CCL5, GDF15 and EDN3. N = 6. (O,P) Representative images and quantitative analysis of SA-β-gal staining in LPS-induced mice models compared with the Sham group. Scale bar = 20 μm, N = 3. (Q,R) Representative Western blot bands and quantitative analysis of P16, P21, P53, and γ-H2AX in CLP-induced mice models compared with the Sham group. N = 6. (S) The mRNA levels of P16, P21, P53 in CLP-induced mouse models compared with the Sham group were detected using qRT-PCR. N = 4. (T) The mRNA levels of SASP-related genes were detected using qRT-PCR in CLP-induced mouse models compared with the Sham group, including IL-1β, IL-6, TNF-α, CXCL1, CXCL3, CCL2, CCL5, GDF15, and TGFβ2. N = 4. (U,V) Representative fluorescence images and quantitative analysis of P21 with cTnI and DAPI in CLP-induced mice models compared with the Sham group. Scale bar = 20 μm, N = 3. Data are presented as mean ± SD. * P<0.05, ** P<0.01, *** P<0.001, **** P<0.0001. SA-ß-gal, senescence-associated β-galactosidase; LPS, Lipopolysaccharide; CLP, cecal ligation and puncture; NRCMs, neonatal rat cardiomyocytes; SASP, senescence-associated secretory phenotype.

To further validate these findings, we conducted *in vitro* tests using NRCMs and *in vivo* experiments involving two animal models, namely the LPS and CLP paradigms. **Figure 1A-B** demonstrated that following a 12-hour co-culture with LPS, we observed a substantial increase in the protein levels of senescence markers, specifically P16, P21, and P53, in NRCMs compared to the control group. Furthermore, the levels of γ-H2AX, a biomarker associated with DNA damage and cellular senescence, were notably increased following LPS stimulation. A consistent and continuous increase in mRNA levels of P16, P21, and P53 was also detected in LPS-induced NRCMs compared with the control group (**Figure 1C**). The findings from the immunofluorescence analysis revealed a considerable rise in P16 levels following LPS stimulation. Additionally, P16 was observed to co-localize with the nuclear marker DAPI in NRCMs (**Figure 1D-E**). The SA-β-gal staining revealed a notable rise in the percentage of NRCMs that exhibited positive staining following LPS stimulation (**Figure 1F-G**). Furthermore, we have provided evidence that the stimulation of LPS led to the induction of SASP in NRCMs, which was supported by the observed increase in mRNA levels of IL-1β, IL-6, TNF-α, CXCL1, CXCL3, CXCL10, CCL2, CCL5, and GDF15 (**Figure 1H**). These findings provided confirmation that LPS had the ability to promote premature senescence and the SASP in cardiomyocytes at the cellular level.

Comparable findings were observed in mouse models of SCM caused by LPS and CLP. The data presented in **Figure 1I-J** demonstrated that LPS-induced mice exhibited elevated protein levels of P16, P21, P53, and γ-H2AX in comparison to the Sham group. Comparable findings were seen with respect to the mRNA levels of P16, P21, and P53** (Figure 1K)**. As displayed in **Figure 1L-M**, the immunofluorescence results demonstrated an elevation in P16 levels in the hearts of mice that were stimulated by LPS.

Most importantly, P16 exhibited a strong co-localization with the cardiomyocyte marker cTnI. Furthermore, the administration of LPS resulted in a substantial upregulation of SASP-related mRNA levels in the cardiac tissue of mice, including IL-1β, IL-6, TNF-α, CXCL1, CXCL3, CCL2, CCL5, GDF15, and EDN3 (**Figure 1N**). The SA-β-gal staining showed a significant increase in the region of heart tissues that displayed positive staining after LPS stimulation compared to the Sham group (**Figure 1O-P**). In CLP-induced mice, protein levels of senescence markers, including P16, P21, P53, and γ-H2AX were also remarkably elevated compared to the Sham group** (Figure 1Q-R)**. The mRNA levels of P16, P21, and P53 exhibited a comparable pattern, as depicted in **Figure 1S**. Moreover, the mRNA levels of SASP-related genes, including IL-1β, IL-6, TNF-α, CXCL1, CXCL3, CCL2, CCL5, GDF15, and TGFβ2, were considerably increased in the mouse hearts of CLP group compared to the Sham group (**Figure 1T**). Co-staining of cTnI and P21 revealed a notable increase in the fluorescence intensity of P21 within the cardiac nucleus following CLP (**Figure 1U-V**). In summary, the available evidence indicated that cardiomyocyte senescence occurred in the SCM mouse models, accompanied by a notable increase of SASP.

### RNA-seq and bioinformatics analysis reveal the potential effects of Rux treatment on mouse hearts

**Figure 2:**
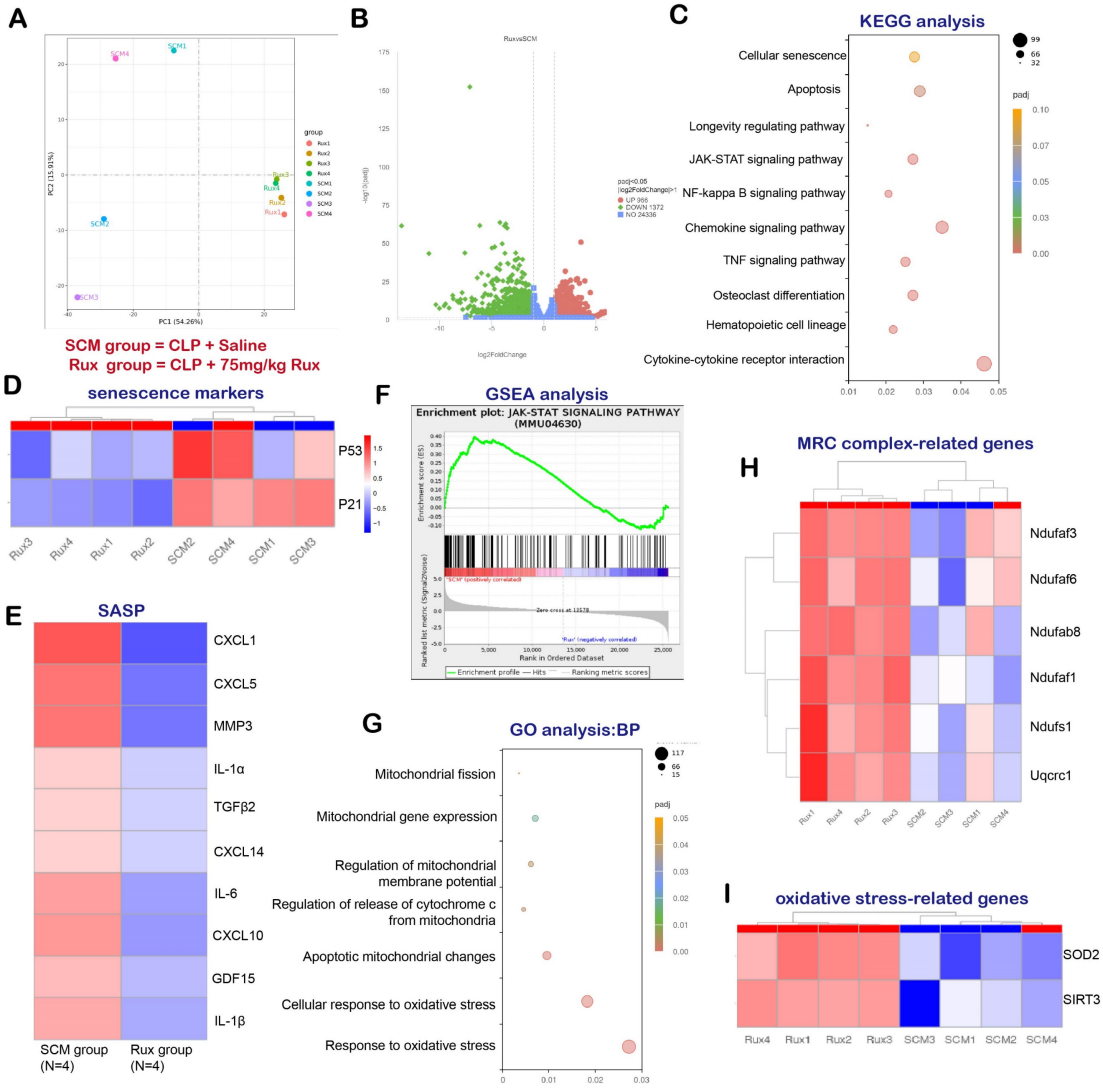
** RNA-seq and bioinformatics analysis reveal the potential effects of Rux treatment on mouse hearts.** (A) PCA identified the mice as two groups, namely the SCM group and the Rux group. N = 4 in each group. (B) DEGs between the SCM group and the Rux treatment group were shown in the volcano map. (C) KEGG analysis identified changes in the cardiac pathways of mice with SCM following Rux therapy. (D) The heat map indicated that senescence markers, specifically P53 and P21, were downregulated in mice hearts with SCM after Rux treatment. N = 4 in each group. (E) The relative levels of SASP-related genes were displayed in the heatmap between the SCM and the Rux groups, including CXCL1, CXCL5, MMP3, IL-1α, TGFβ2, CXCL14, IL-6, CXCL10, GDL15, and IL-1β. N = 4 in each group. (F) GSEA analysis demonstrated that the JAK-STAT pathway was up-regulated in SCM-induced mouse hearts and down-regulated after treatment with Rux. (G) GO analysis identified changes in the cardiac biological processes of SCM mice following Rux therapy. (H) The heatmap demonstrated that the relative levels of MRC complex-related genes were upregulated post Rux treatment in SCM-induced mice hearts. N = 4 in each group. (I) The heatmap demonstrated that the relative levels of oxidative stress-related genes were up-regulated post Rux treatment in SCM-induced mice hearts. N = 4 in each group. Data are presented as mean ± SD. * P<0.05, ** P<0.01, *** P<0.001, **** P<0.0001. KEGG, Kyoto Encyclopedia of Genes and Genomes; GSEA, Gene Set Enrichment Analysis; MRC, mitochondrial respiratory chain; SASP, senescence-associated secretory phenotype; GO, Gene Ontology; Rux, ruxolitinib; SCM, septic cardiomyopathy; DEGs, differentially expressed genes.

In order to examine the potential of Rux-based senomorphic therapy in mitigating SCM-induced cardiac senescence in mice, we conducted RNA-seq and a variety of bioinformatics procedures on two distinct groups of mice: SCM mice treated with saline and SCM mice treated with Rux. As shown in **Figure 2A**, Principal Component Analysis (PCA) revealed that mice subjected to two different treatments were segregated into two distinct groups, exhibiting no substantial overlap. The volcano map in **Figure 2B** displayed DEGs, with 966 genes exhibiting significant upregulation and 1372 genes exhibiting significant downregulation following Rux therapy in hearts of mouse models. The findings from the KEGG analysis in **Figure 2C** indicated notable alterations in cellular senescence, the longevity regulating pathway, and the JAK-STAT pathway subsequent to Rux therapy. Additionally, the heat map demonstrated that SCM mice subjected to Rux therapy had a notable decrease in senescence markers in hearts, such as P21 and P53, in comparison to the group treated with saline (**Figure 2D**). As displayed in **Figure 2E**, after Rux therapy, the mRNA levels of SASP-associated genes, such as CXCL1, CXCL5, MMP3, IL-1α, TGFβ2, CXCL14, IL-6, CXCL10, GDL15, and IL-1β, were dramatically suppressed. GSEA analysis provided additional evidence supporting the activation of the JAK-STAT pathway following SCM, whereas Rux therapy had inhibitory effects on its activity (**Figure 2F**). GO analysis demonstrated that Rux therapy had a significant impact on various biological processes associated with mitochondrial involvement, including mitochondrial fission, mitochondrial gene expression, regulation of mitochondrial membrane potential, regulation of cytochrome c (Cyto-c) release from mitochondria, apoptotic mitochondrial changes, cellular response to oxidative stress, and response to oxidative stress (**Figure 2G**). **Figure 2H** demonstrated that genes associated with MRC complex I, such as Ndufaf3, Ndufaf6, Ndufab8, Ndufaf1, Ndufs1, and MRC complex III genes like Uqcrc1, exhibited an upward trend in expression following Rux therapy in mouse hearts. Furthermore, it was worth noting that crucial genes implicated in oxidative stress, namely SOD2 and SIRT3, were also involved in Rux-mediated therapeutic mechanisms in the context of SCM (**Figure 2I**). Based on the findings presented, it is postulated that Rux treatment exhibited promise in mitigating cardiac senescence generated by SCM and ameliorating mitochondrial dysfunction and oxidative stress induced by SCM, which might be mediated by the JAK-STAT pathway.

### Senomorphic therapy with Rux exerts an anti-SASP effect and attenuates LPS-induced cellular senescence in NRCMs

**Figure 3:**
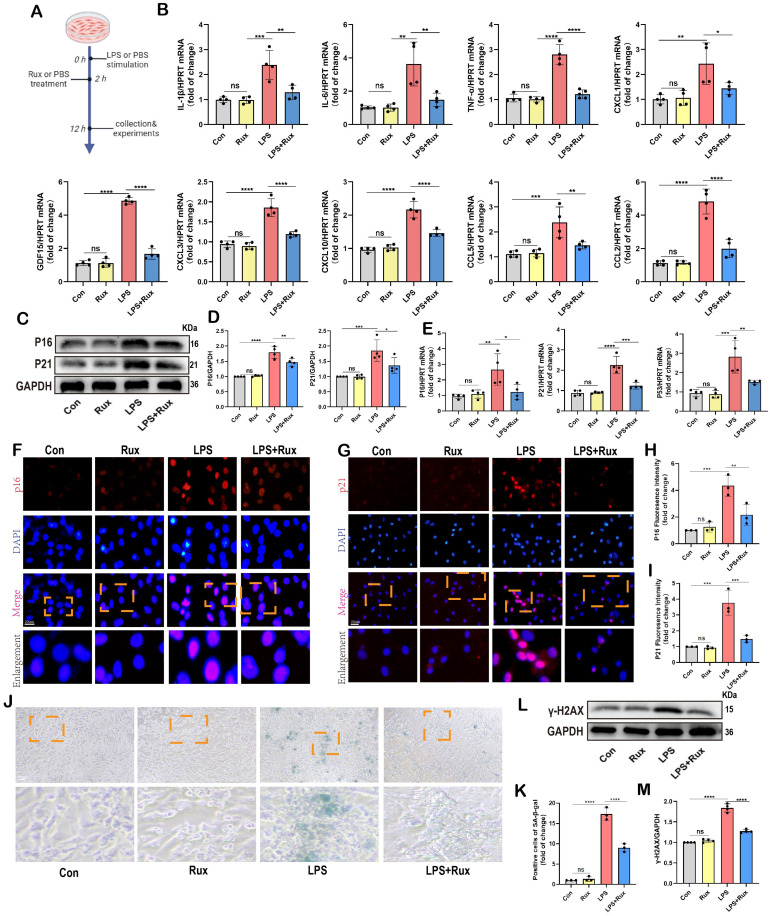
** Senomorphic therapy with Rux exerts an anti-SASP effect and attenuates LPS-induced cellular senescence in NRCMs.** (A) Schematic diagram of *in vitro* experiments in NRCMs. (B) The mRNA levels of SASP-related genes were detected using qRT-PCR in NRCMs treated with Rux or not post LPS-stimulation, including IL-1β, IL-6, TNF-α, CXCL1, CXCL3, CXCL10, CCL2, CCL5 and GDF15. N = 4. (C,D) Representative Western blot bands and quantitative analysis of P16 and P21 in NRCMs treated with Rux or not post LPS stimulation compared with the control group. N = 4. (E) The mRNA levels of P16, P21, and P53 were detected using qRT-PCR in NRCMs treated with Rux or not post LPS stimulation compared with the control group. N = 4. (F-I) Representative fluorescence images and quantitative analysis of P16 and P21 with DAPI in NRCMs treated with Rux or not post LPS stimulation. Scale bar = 20 μm, N = 3. (J,K) Representative images and quantitative analysis of SA-β-gal staining in NRCMs treated with Rux or not post LPS stimulation. Scale bar = 20 μm, N = 3. (L,M) Representative Western blot bands and quantitative analysis of γ-H2AX in NRCMs treated with Rux or not post LPS stimulation. N = 4. Data are presented as mean ± SD. * P<0.05, ** P<0.01, *** P<0.001, **** P<0.0001. Rux, ruxolitinib; Con, control; LPS, Lipopolysaccharide; SASP, senescence-associated secretory phenotype; NRCMs, neonatal rat cardiomyocytes.

Initially, we conducted *in vitro* experiments in LPS-induced NRCMs to investigate whether senomorphic treatment with Rux could generate an anti-SASP effect and subsequently alleviate cellular senescence in SCM. As displayed in **Figure 3A**, in the Rux treatment group, Rux was added 2 hours after LPS stimulation to achieve a final concentration of 1μM in the culture medium, and co-cultured until 12 hours. In NRCMs, we observed that treatment with Rux did not alter the transcription levels of SASP-related genes under physiological conditions, but that LPS stimulation significantly induced SASP, and Rux treatment reversed the increase in mRNA levels of these genes (IL-1β, IL-6, TNF-α, CXCL1, CXCL3, CXCL10, CCL2, CCL5, and GDF15), as illustrated in **Figure 3B**. More importantly, the Rux therapy effectively counteracted the LPS-induced rise in the protein levels of the senescence markers P16 and P21 (**Figure 3C-D**). Similar results were noted for mRNA levels of P16, P21, and P53, which were upregulated following LPS stimulation and suppressed by Rux administration (**Figure 3E**). The fluorescence intensity of P16 and P21 in the nucleus of NRCMs increased significantly in response to LPS stimulation, as seen by immunofluorescence staining, and Rux administration brought the fluorescence intensity back to levels comparable to those of the control group (**Figure 3F-I**). As shown in **Figure 3J-K**, the anti-senescence efficacy of Rux on LPS-induced NRCMs was further validated by SA-β-gal staining, demonstrating a significant reduction in the percentage of positively stained cells following Rux therapy. Furthermore, as displayed in **Figure 3L-M**, Rux treatment did not exhibit DNA damage toxicity under physiological conditions, but it did attenuate LPS-induced DNA damage in cardiomyocytes and reverse the up-regulation of γ-H2AX expression level that induced by LPS. Additionally, we further validated the therapeutic efficacy of Rux in cell models induced by H_2_O_2_ and D-gal. Treatment with Rux significantly suppressed the H_2_O_2_-induced protein expression of P16 and P53, indicating a reduction in cellular senescence (**Figure S2A**). Consistently, mRNA levels of senescence markers P16, P21, and P53 corroborated these protein-level changes (**Figure S2B**). Moreover, following H_2_O_2_ stimulation, the expression of SASP factors, including CXCL1, CXCL3, CXCL10, TNF-α, IL-6, and EDN3, were elevated, but Rux treatment effectively attenuated their induction (**Figure S2C**). Similar outcomes were observed in D-galactose-induced cell models, where Rux therapy significantly downregulated the expression of senescence markers induced by D-galactose at both the protein and mRNA levels (**Figure S2D-F**). Based on this data, Rux might have a potent cellular anti-SASP impact and ultimately alleviate cardiomyocyte cellular senescence, providing a novel therapeutic perspective for SCM.

### Senomorphic therapy with Rux exerts an anti-SASP effect and attenuates cellular senescence in mouse models with SCM

**Figure 4:**
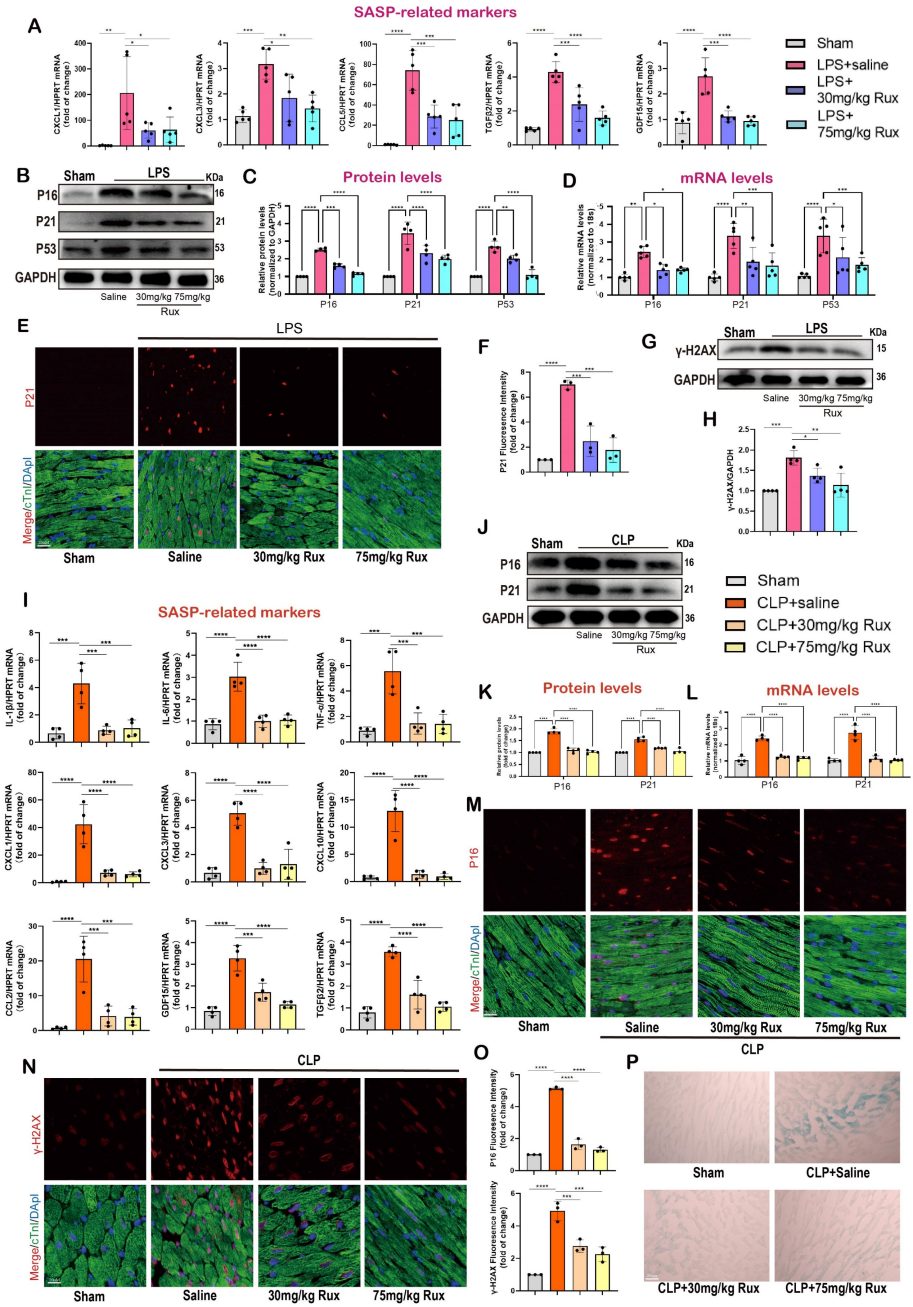
** Senomorphic therapy with Rux exerts an anti-SASP effect and attenuates cellular senescence in mouse models with SCM.** (A) The mRNA levels of SASP-related genes were detected using qRT-PCR in mouse hearts treated with 30 mg/kg and 75 mg/kg of Rux or saline post LPS-injection compared with the Sham group, including CXCL1, CXCL3, CCL5, TGFβ2 and GDF15. N = 4. (B,C) Representative Western blot bands and quantitative analysis of P16, P21, and P53 in mouse hearts treated with 30 mg/kg and 75 mg/kg of Rux or saline post LPS injection compared with the Sham group. N = 4. (D) The mRNA levels of P16, P21, and P53 were detected using qRT-PCR in mouse hearts treated with 30 mg/kg and 75 mg/kg of Rux or saline post LPS injection compared with the Sham group. N = 4. (E,F) Representative fluorescence images and quantitative analysis of P21 with cTnI and DAPI in mouse hearts treated with 30 mg/kg and 75 mg/kg of Rux or saline post LPS injection compared with the Sham group. Scale bar = 20 μm, N = 3. (G,H) Representative Western blot bands and quantitative analysis of γ-H2AX in mouse hearts treated with 30 mg/kg and 75 mg/kg of Rux or saline post LPS injection compared with the Sham group. N = 4. (I) The mRNA levels of SASP-related genes were detected using qRT-PCR in mouse hearts treated with 30 mg/kg and 75 mg/kg Rux or saline post CLP-surgery compared with the Sham group, including IL-1β, IL-6, TNF-α, CXCL1, CXCL3, CXCL10, CCL2, TGFβ2, and GDF15. N = 4. (J,K) Representative Western blot bands and quantitative analysis of P16 and P21 in mouse hearts treated with 30 mg/kg and 75 mg/kg of Rux or saline post CLP-surgery compared with the Sham group. N = 4. (L) The mRNA levels of P16 and P21 were detected using qRT-PCR in mouse hearts treated with 30 mg/kg and 75 mg/kg of Rux or saline post CLP-surgery compared with the Sham group. N = 4. (M-O) Representative fluorescence images and quantitative analysis of P21 and γ-H2AX with cTnI and DAPI in mouse hearts treated with 30 mg/kg and 75 mg/kg of Rux or saline post CLP-surgery compared with the Sham group. Scale bar = 20 μm, N = 3. (P) Representative images of SA-β-gal staining in mouse hearts treated with 30 mg/kg and 75 mg/kg of Rux or saline post CLP-surgery compared with the Sham group. Scale bar = 20 μm. N=3. Data are presented as mean ± SD. * P<0.05, ** P<0.01, *** P<0.001, **** P<0.0001. SA-ß-gal, senescence-associated β-galactosidase; LPS, Lipopolysaccharide; CLP, cecal ligation and puncture; SASP, senescence-associated secretory phenotype; Rux, ruxolitinib.

Given the excellent anti-senescence effect of Rux therapy in *in vitro* experiments, we further validated its effects in LPS- and CLP-induced mouse models. The **Figure 4A** demonstrated that both low and high-dose Rux therapy effectively reduced the mRNA levels of CXCL1, CXCL3, CCL5, TGFβ2, and GDF15 in cardiac tissues of LPS-induced mouse models, indicating strong anti-SASP actions. As displayed in **Figure 4B-C**, the Western blot analysis demonstrated that the elevated expression of P16, P21, and P53 proteins in the cardiac tissues of mice induced by LPS was mitigated by the administration of Rux in both the low and high-dose groups. It is important to mention that administering high doses of Rux does not provide any additional benefits compared to low-dose treatment. Subsequently, to ascertain the presence of cellular senescence in cardiomyocytes, P21 and cTnI were simultaneously labeled with fluorescence. As shown in **Figure 4E-F**, our findings indicated that LPS notably increased the expression of P21 in the nucleus of cardiomyocytes, however, both low and high dosages of Rux administration were able to decrease the fluorescence intensity of P21 in the nucleus of cardiomyocytes. Moreover, as shown in **Figure 4G-H**, the DNA of the mouse heart experienced significant damage when exposed to LPS, as indicated by an increase in γ-H2AX levels. However, the administration of low and high doses of Rux resulted in a decrease in γ-H2AX protein expression levels. These results indicated that Rux treatment could effectively mitigate DNA damage and cellular senescence in SCM.

The CLP-induced mouse model is a widely utilized paradigm for replicating the pathophysiological mechanisms of SCM. We conducted additional verification to confirm the preventive effect of Rux-based senomorphic therapy on SCM using CLP-induced models. As depicted in **Figure 4I**, following CLP surgery, the levels of SASP-related cytokines and chemokines were consistently elevated while treatment with low and high doses of Rux within 2 and 6 hours after CLP surgery effectively reversed the expression levels of these mRNA levels, including IL-1β, IL-6, TNF-α, CXCL1, CXCL3, CXCL10, CCL2, TGFβ2, and GDF15. Not surprisingly, Rux effectively reduced the cardiac senescence induced by CLP, as evidenced by the decrease in protein and mRNA levels of P16 and P21 (**Figure 4J-L**). As shown in **Figure 4M-O**, the immunofluorescence results demonstrated that CLP caused a significant increase in the levels of P16 and γ-H2AX in the nuclei of cardiomyocytes in the heart.

However, treatment with 30 or 75mg/kg of Rux reduced the expression of these two markers in cardiomyocytes of CLP-induced mouse models, as confirmed by the co-localization of cTnI and P16 or γ-H2AX. More importantly, the SA-β-gal staining further confirmed that Rux treatment could alleviate the senescence of cardiac tissue (**Figure 4P, Figure S3**). Overall, our results provided solid evidence at the animal level based on two authoritative animal models, suggesting that low doses of Rux (30 mg/kg) were sufficient to reduce cardiomyocyte senescence in the hearts of mice with SCM.

### Senomorphic therapy with Rux alleviates cardiac dysfunction and mitochondrial-mediated apoptosis in mice with SCM

**Figure 5:**
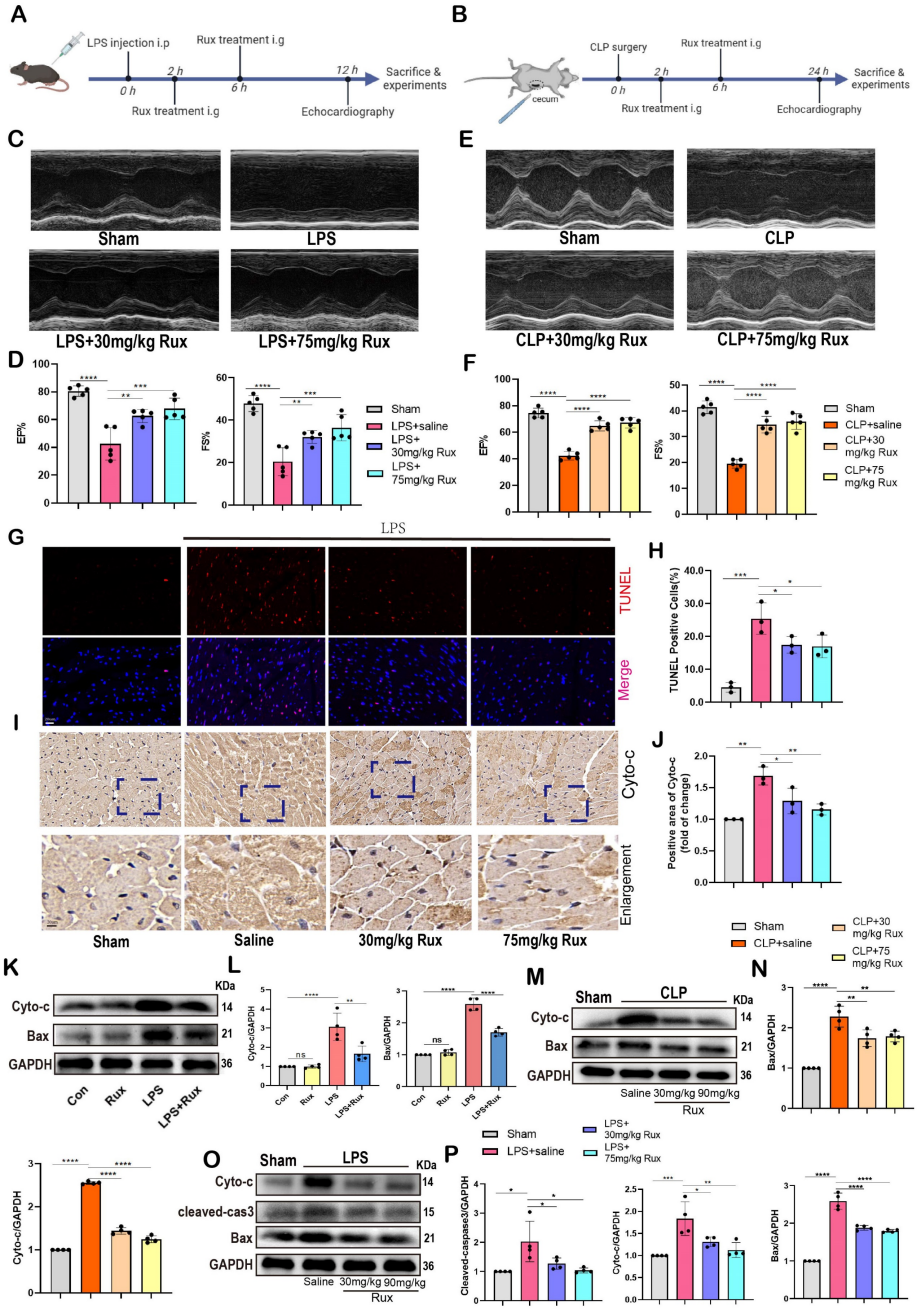
** Rux treatment alleviates cardiac dysfunction and mitochondrial-mediated apoptosis in mice with SCM.** (A) Schematic diagram of *in vivo* experiments in LPS-induced mouse models. (B) Schematic diagram of *in vivo* experiments in CLP-induced mouse models. (C,D) Cardiac function indices were measured by echocardiography in mice treated with 30 mg/kg and 75 mg/kg of Rux or saline post LPS injection compared with the Sham group. N = 5. (E,F) Cardiac function indices were measured by echocardiography in mice treated with 30 mg/kg and 75 mg/kg of Rux or saline post CLP surgery compared with the Sham group. N = 5. (G,H) Representative images and quantitative analysis of TUNEL assays in in mouse hearts treated with 30 mg/kg and 75 mg/kg of Rux or saline post LPS injection compared with the Sham group. Scale bar = 20 μm, N = 3. (I,J) Representative immunohistochemistry images and quantitative analysis of Cyto-c expression levels in in mouse hearts treated with 30 mg/kg and 75 mg/kg of Rux or saline post LPS injection compared with the Sham group. Scale bar = 20 μm, N = 3. (K,L) Representative Western blot bands and quantitative analysis of cyto-c and Bax in in NRCMs treated with Rux or not post LPS stimulation. N = 4. (M,N) Representative Western blot bands and quantitative analysis of cyto-c and Bax in mouse hearts treated with 30 mg/kg and 75 mg/kg of Rux or saline post CLP surgery compared with the Sham group. N = 4. (O,P) Representative Western blot bands and quantitative analysis of cyto-c, cleaved-cas3 and Bax in mouse hearts treated with 30 mg/kg and 75 mg/kg Rux or saline post LPS injection compared with the Sham group. N = 4. Data are presented as mean ± SD. * P<0.05, ** P<0.01, *** P<0.001, **** P<0.0001. LPS, Lipopolysaccharide; CLP, cecal ligation and puncture; Rux, ruxolitinib; EF%, ejection fraction; FS%, fractional shortening; Cyto-c, Cytochrome c; Con, control; NRCMs, neonatal rat cardiomyocytes.

As displayed in **Figure 5A-B**, to investigate the potential of Rux therapy in improving cardiac dysfunction in mice with SCM, we assessed cardiac function-related parameters using echocardiography in mouse models induced by LPS and CLP. As shown in **Figure 5C-F**, the results of echocardiography revealed that the injection of LPS and the performance of CLP surgery led to a notable suppression of cardiac contractility in mice, as indicated by reduced EF% and FS%. Conversely, the administration of both low and high doses of Rux resulted in a significant enhancement of cardiac contractility. These results indicated that Rux therapy had great potential to improve the decreased cardiac contractility caused by SCM. Furthermore, as displayed in **Figure 5 G-H**, TUNEL assay revealed that LPS significantly induced cellular apoptosis in the mouse hearts, as evidenced by an increase in positively stained cells, while treatment with both 30 mg and 75 mg/kg of Rux could reverse this effect. Furthermore, we investigated whether low-dose Rux treatment could restore heart function after SCM was established (**Figure S4A**). As depicted in **Figure S4B-C**, we observed that 12 hours after CLP surgery, heart function was significantly impaired, as indicated by EF% and FS%. However, treatment with 30 mg/kg of Rux at 12 hours post CLP surgery reversed the decline in heart function by 24 hours compared to the saline-treated group.

A recent study demonstrated that sublethal mitochondrial apoptotic stress is a major driver of the SASP and cellular senescence^34^. The immunohistochemical results showed that both low and high doses of Rux treatment inhibited the upregulation of Cyto-C expression in LPS-induced mouse hearts, suggesting that the enhanced mitochondrial apoptotic stress could be inhibited by Rux (**Figure 5I-J**). Accordingly, the levels of mitochondrial mediated-apoptosis related proteins were detected in this study. In **Figure 5K-L**, our findings from *in vitro* experiments demonstrated that Rux therapy effectively inhibited the increase in protein levels of Cyto-C and Bax induced by LPS in NRCMs. Importantly, Rux had no impact on these protein levels under normal physiological conditions. We consistently observed a considerable activation of the mitochondrial-mediated apoptotic pathway in mouse models produced by CLP, as evidenced by an increase in protein levels of Cyto-C and Bax, and both low-dose and high-dose Rux treatments were effective in reversing this trend (**Figure 5M-N**). Furthermore, LPS-induced models were utilized to validate these findings. As shown in **Figure 5O-P**, consistent with expectations, the administration of Rux therapy resulted in a decrease in the protein levels of Cyto-C and Bax in mouse hearts following LPS injection, regardless of the dosage, compared to the saline treatment group.

To summarize, these findings suggested that Rux treatment might be an effective strategy for enhancing cardiac dysfunction caused by sepsis, and reducing mitochondrial-mediated apoptotic stress might be one of the underlying mechanisms for alleviating cellular senescence.

### Senomorphic therapy with Rux alleviates oxidative stress and mitochondrial dysfunction in SCM

Oxidative stress is a major factor in the progression of SCM and cellular senescence, potentially due to mitochondrial damage caused by ROS^13^, ^14^.

**Figure 6:**
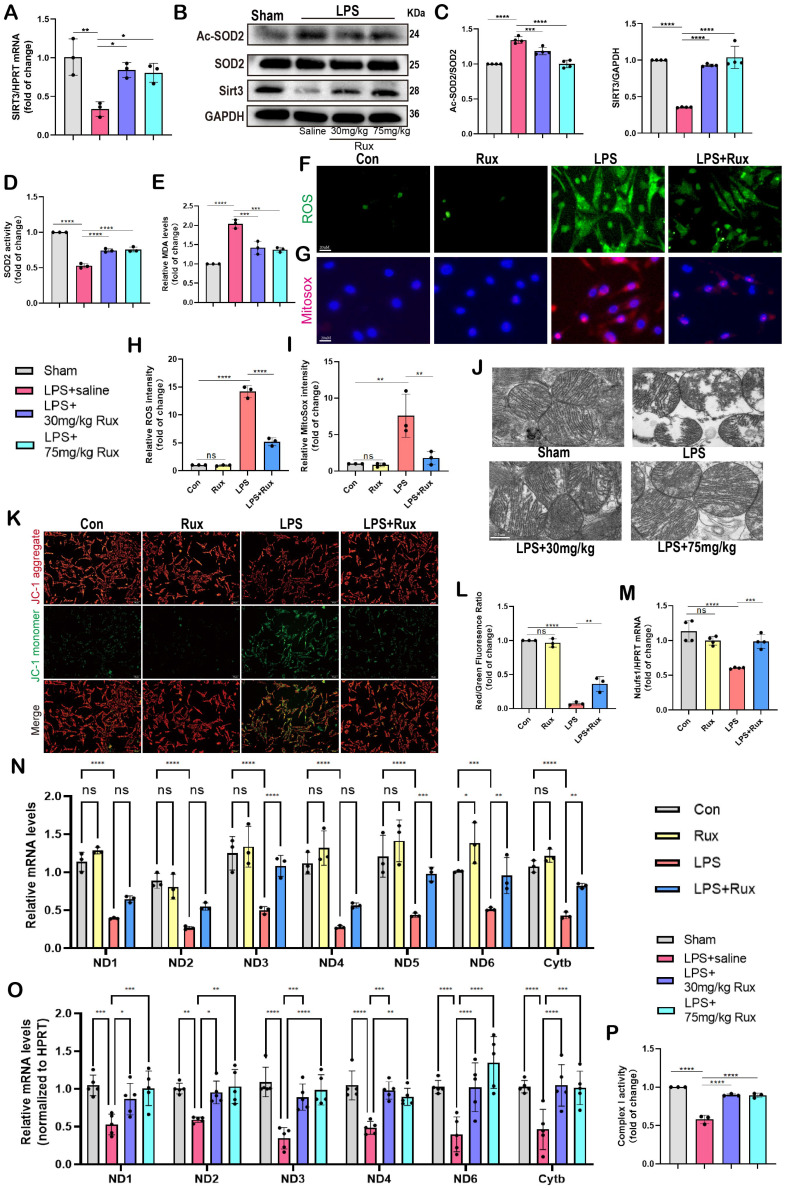
** Senomorphic therapy with Rux alleviates oxidative stress and mitochondrial dysfunction in SCM.** (A) The mRNA levels of Sirt3 were detected using qRT-PCR in mouse hearts treated with 30 mg/kg and 75 mg/kg of Rux or saline post LPS injection compared with the Sham group. N = 3. (B,C) Representative Western blot bands and quantitative analysis of Ac-SOD2, SIRT3 and SOD2 in mouse hearts treated with 30 mg/kg and 75 mg/kg of Rux or saline post LPS injection compared with the Sham group. N = 4. (D) Relative SOD2 activity in mouse hearts treated with 30 mg/kg and 75 mg/kg of Rux or saline post LPS injection compared with the Sham group. N = 3. (E) Relative MDA levels in mouse hearts treated with 30 mg/kg and 75 mg/kg of Rux or saline post LPS injection compared with the Sham group. N = 3. (F-I) Representative fluorescence images and quantitative analysis of ROS and MitoSox in NRCMs treated with Rux or not post LPS stimulation. Scale bar = 20 μm, N =3. (J) Representative TEM images of mitochondria in mouse hearts treated with 30 mg/kg and 75 mg/kg of Rux or saline post LPS injection compared with the Sham group. Scale bar = 0.5 μm. (K,L) Representative fluorescence images and quantitative analysis of JC-1 staining in NRCMs treated with Rux or not post LPS stimulation. Scale bar = 20 μm, N = 3. (M) The mRNA levels of Ndufs1 were detected using qRT-PCR in NRCMs treated with Rux or not post LPS stimulation. N = 4. (N) The mRNA levels of MRC complex-related genes were detected using qRT-PCR in NRCMs treated with Rux or not post LPS stimulation. N = 3. (O) The mRNA levels of MRC complex-related genes were detected using qRT-PCR in mouse hearts treated with 30 mg/kg and 75 mg/kg of Rux or saline post LPS injection compared with the Sham group. N = 4. (P) The relative MRC complex I activity was detected in mouse hearts treated with 30 mg/kg and 75 mg/kg of Rux or saline post LPS injection compared with the Sham group. N = 3. Data are presented as mean ± SD. * P<0.05, ** P<0.01, *** P<0.001, **** P<0.0001. LPS, Lipopolysaccharide; Rux, ruxolitinib; Con, control; ND, NADH dehydrogenase; Cytb, cytochrome b; NRCMs, neonatal rat cardiomyocytes.

Therefore, we investigated whether treatment with Rux could reduce oxidative stress in cardiomyocytes in the context of SCM. Remarkably, we discovered that the administration of 30 mg/kg and 75 mg/kg of Rux restored the decrease in Sirt3 mRNA levels in mouse hearts caused by LPS (**Figure 6A**). It was reported that Sirt3 impairment reduced the activity of a key mitochondrial antioxidant enzyme, superoxide dismutase 2 (SOD2) because of hyperacetylation^35^.

Accordingly, as shown in **Figure 6B-C**, our findings indicated that the protein levels of Sirt3 were noticeably reduced in mouse hearts stimulated with LPS. However, treatment with both low and high doses of Rux restored these protein levels. Consistently, the levels of acetylated SOD2 protein exhibited an inverse pattern compared to the changes in Sirt3 protein (**Figure 6B-C**). Subsequently, we assessed the enzymatic activity of SOD2 and observed that treatment with low and high doses of Rux reversed the decrease in SOD2 activity induced by LPS (**Figure 6D**). In contrast, the levels of MDA were notably increased in the hearts of mice treated with LPS, indicating the presence of oxidative stress, which was alleviated by treatment with 30 and 75 mg/kg of Rux (**Figure 6E**). As depicted in **Figure 6F-I**, the DCFH-DA assay and MitoSox staining revealed that LPS effectively triggered the production of ROS in cardiomyocytes and mitochondria in NRCMs. However, treatment with Rux dramatically decreased the fluorescence intensity of ROS and MitoSox. These findings indicated that both low and high-dose Rux therapy could rebalance the antioxidant system and ROS in SCM, leading to a reduction in oxidative stress.

Based on the results from TEM in **Figure 6J**, it was observed that the mitochondrial structure in the mouse heart attacked by LPS was severely impaired, as evidenced by the destruction of ridges and an increase in vacuoles. Notably, 30 mg/kg and 75 mg/kg of Rux treatment effectively reduced the damage to the mitochondrial structure. JC-1 is an optimal fluorescent indicator for the detection of mitochondrial membrane potential (MMP). The reduction in MMP can be readily identified by the transition of JC-1 fluorescence from red to green, which can also serve as an early signal for detecting apoptosis. The **Figure 6K-L** demonstrated that a considerable decrease in the MMP of NRCMs following LPS stimulation, as indicated by a downregulation of the red/green fluorescence ratio. However, treatment with Rux considerably increased the red/green fluorescence ratio after LPS stimulation.

As shown in **Figure 6N**, the mRNA levels of genes associated with subunits of the MRC complex I and III were noticeably reduced in NRCMs attacked by LPS. However, treatment with Rux restored the mRNA levels of ND3, ND5, ND6, and Cytb. Similar results were obtained in LPS-attacked mouse hearts, where LPS assault strongly down-regulated mRNA levels of ND1, ND2, ND3, ND4, ND6, and Cytb in mouse hearts, and both low and high dosages of Rux therapy completely reversed this trend (**Figure 6O**). More importantly, we noticed that the administration of low and high dosages of Rux restored the functionality of MRC complex I, which had been impaired during SCM (**Figure 6P**). Taken together, it could be inferred that senomorphic treatment with Rux could decrease oxidative stress and restore mitochondrial function in SCM, which might be a significant mechanism by which Rux treatment reduced cellular senescence.

### Senomorphic therapy with Rux restores mitochondrial dynamics and alleviated endoplasmic reticulum stress in SCM

**Figure 7:**
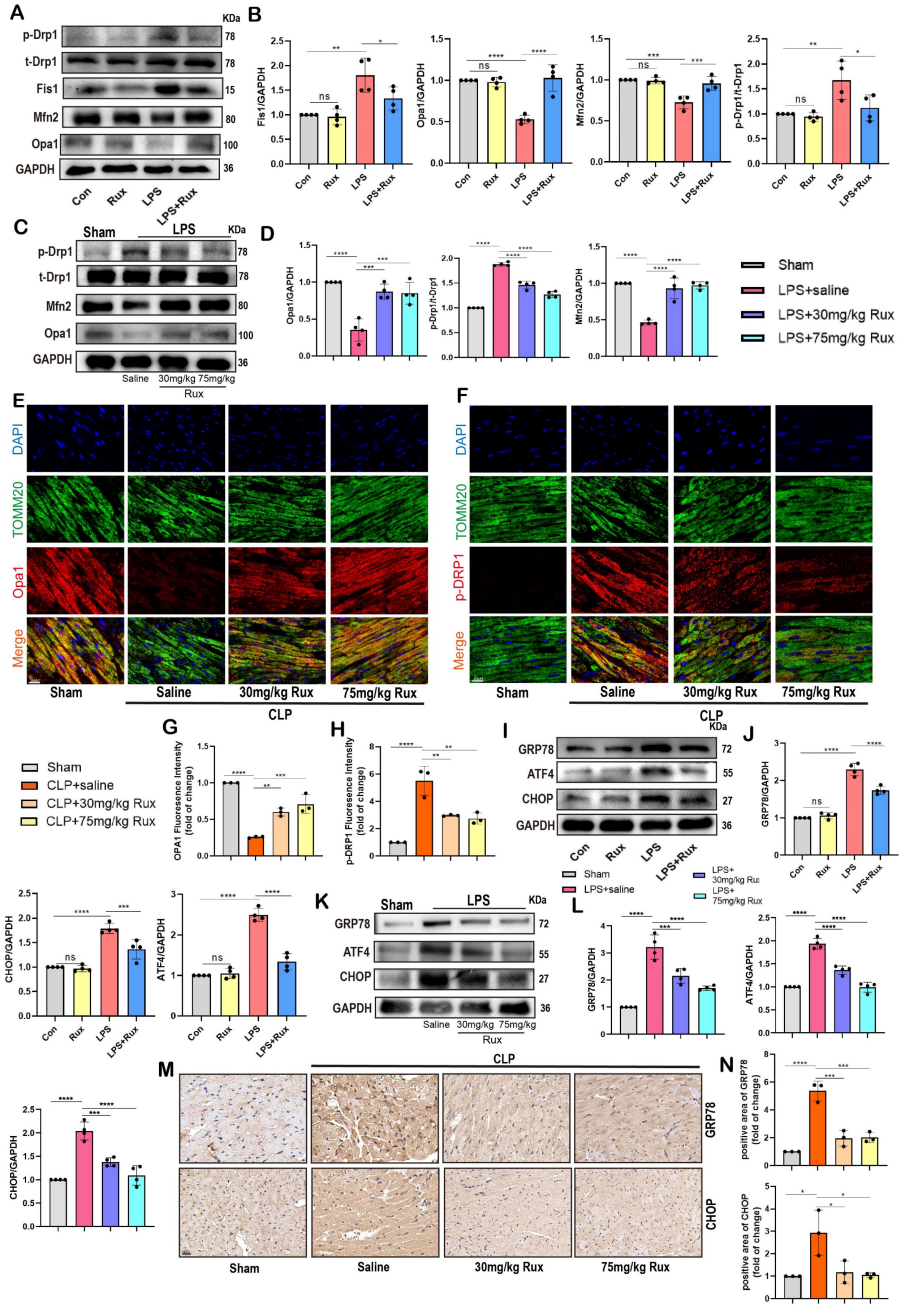
** Senomorphic therapy with Rux restores mitochondrial dynamics and alleviated endoplasmic reticulum stress in SCM.** (A,B) Representative Western blot bands and quantitative analysis of p-Drp1, Fis1, Mfn2 and Opa1 in NRCMs treated with Rux or not post LPS stimulation compared with the control group. N = 4. (C,D) Representative Western blot bands and quantitative analysis of p-Drp1, Mfn2 and Opa1 in mouse hearts treated with 30 mg/kg and 75 mg/kg of Rux or saline post LPS injection compared with the Sham group. N = 4. (E-H) Representative fluorescence images and quantitative analysis of Opa1 and p-Drp1 with TOMM20 and DAPI in mouse hearts treated with 30 mg/kg and 75 mg/kg of Rux or saline post CLP surgery compared with the Sham group. Scale bar = 20 μm, N = 3. (I,J) Representative Western blot bands and quantitative analysis of GRP78, ATF4 and CHOP in NRCMs treated with Rux or not post LPS stimulation compared with the control group. N = 4. (K,L) Representative Western blot bands and quantitative analysis of GRP78, ATF4 and CHOP in mouse hearts treated with 30 mg/kg and 75 mg/kg of Rux or saline post LPS injection compared with the Sham group. N = 4. (M,N) Representative immunohistochemistry images and quantitative analysis of GRP78 and CHOP expression levels in in mouse hearts treated with 30 mg/kg and 75 mg/kg of Rux or saline post CLP surgery compared with the Sham group. Scale bar = 20 μm, N = 3. Data are presented as mean ± SD. * P<0.05, ** P<0.01, *** P<0.001, **** P<0.0001. LPS, Lipopolysaccharide; CLP, cecal ligation and puncture; Rux, ruxolitinib; Con, control; NRCMs, neonatal rat cardiomyocytes.

Restoring dysregulated mitochondrial dynamics has been demonstrated as a useful strategy for alleviating SCM and cellular senescence^30^, ^36^, ^37^. Therefore, we detected proteins associated with mitochondrial fusion and fission to explore if Rux treatment could rescue impaired mitochondrial dynamics. As shown in **Figure 7A-B**, results of the Western blot demonstrated that in NRCMs, LPS stimulation led to excessive mitochondrial fission, as indicated by elevated levels of p-Drp1 and Fis1 proteins. However, treatment with Rux reversed this process. In contrast, the stimulation of LPS suppressed the proteins Opa1 and Mfn2, which are involved in mitochondrial fusion, resulting in a decrease in their expression in NRCMs. Nevertheless, the administration of Rux restored the expression of Opa1 and Mfn2.

As displayed in** Figure 7C-D**, the findings were confirmed in mouse models exposed to LPS. Our data revealed that LPS stimulation resulted in an upregulation of p-Drp1 protein and a downregulation of Opa1 and Mfn2 proteins in the cardiac tissue of mice. However, treatment with low-dose and high-dose Rux restored the expression levels of these proteins. Moreover, we co-stained mitochondrial membrane 20 (Tomm20) with p-Drp1 or Opa1 in the heart tissue of CLP-induced mouse models, as shown in **Figure 7E-F**. Following CLP surgery, we observed a significant increase in the fluorescence intensity of mitochondrial p-Drp1, while the intensity of Opa1 was reduced. However, treatment with 30 and 75 mg/kg of Rux reversed these changes.

Mitofusin 2 (Mfn2) was a mitochondrial membrane protein with a role connecting endoplasmic reticulum (ER) membranes to mitochondria^38^, ^39^, and its depletion caused ER stress^40^, ^41^. It has been shown that ER stress was closely associated with cellular senescence, as indicated by previous research^42^. Hence, we proceeded to analyze ER stress-associated proteins in NRCMs and in animal models induced with LPS and CLP. In **Figure 7I-J**, results demonstrated a significant increase in protein levels of CHOP, GRP78, and ATF4 after LPS stimulation, indicating the occurrence of ER stress in NRCMs, whereas treatment with Rux suppressed the protein expression levels of these proteins. Additionally, we found that Rux treatment had no effects on ER stress compared with the control group under physiological conditions. As shown in **Figure 7K-L**, our results confirmed that treatment with low and high dosages of Rux dramatically decreased the elevated levels of ER stress-related proteins (CHOP, GRP78, and ATF4) in the heart tissue of mice induced by LPS compared to mice treated with saline. Subsequently, we further validated the anti-ER stress effect using immunohistochemical staining in CLP-induced mouse hearts, where treatment with 30 and 75mg/kg of Rux significantly inhibited the upregulation of GRP78 and CHOP induced by CLP** (Figure 7M-N)**.

These findings indicated that restoring mitochondrial dynamics while inhibiting ER stress might be a crucial mechanism by which Rux treatment mitigates cellular senescence during the process of SCM.

### Senomorphic therapy with Rux regulates mitochondrial dynamics and mitochondrial-mediated apoptosis by inhibiting the JAK2-STAT3 signaling pathway

**Figure 8:**
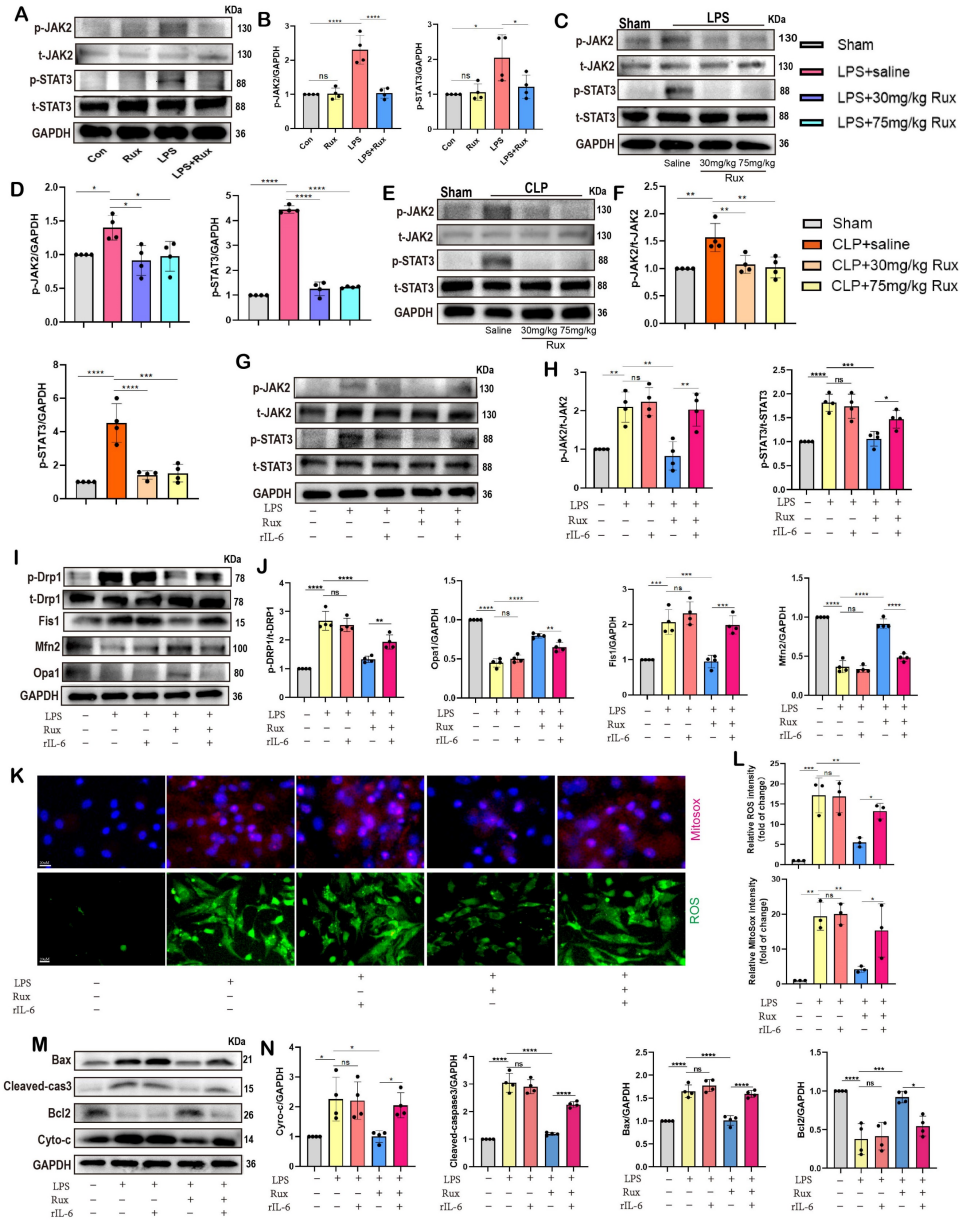
** Senomorphic therapy with Rux regulates mitochondrial dynamics and mitochondrial-mediated apoptosis by inhibiting the JAK2-STAT3 signaling pathway.** (A,B) Representative Western blot bands and quantitative analysis of p-JAK2 and p-STAT3 in NRCMs treated with Rux or not post LPS stimulation compared with the control group. N = 4. (C,D) Representative Western blot bands and quantitative analysis of p-JAK2 and p-STAT3 in mouse hearts treated with 30 mg/kg and 75 mg/kg of Rux or saline post LPS injection compared with the Sham group. N = 4. (E,F) Representative Western blot bands and quantitative analysis of p-JAK2 and p-STAT3 in mouse hearts treated with 30 mg/kg and 75 mg/kg of Rux or saline post CLP surgery compared with the Sham group. N = 4. (G,H) Representative Western blot bands and quantitative analysis of p-JAK2 and p-STAT3 after reactivating the JAK2-STAT3 pathway using r-IL6 in NRCMs. N = 4. (I,J) Representative Western blot bands and quantitative analysis of p-Drp1, Fis1, Mfn2 and Opa1 after reactivating the JAK2-STAT3 pathway using r-IL6 in NRCMs. N = 4. (K,L) Representative fluorescence images and quantitative analysis of ROS and MitoSox after reactivating the JAK2-STAT3 pathway using r-IL6 in NRCMs. Scale bar = 20 μm, N = 4. (M,N) Representative Western blot bands and quantitative analysis of Bax, Cleaved-cas3, Bcl2 and Cyto-c after reactivating the JAK2-STAT3 pathway using r-IL6 in NRCMs. N = 4. Data are presented as mean ± SD. * P<0.05, ** P<0.01, *** P<0.001, **** P<0.0001. LPS, Lipopolysaccharide; Rux, ruxolitinib; Con, control; Cyto-c, Cytochrome c; ROS, reactive oxygen species; r-IL6, recombinant IL-6; NRCMs, neonatal rat cardiomyocytes.

Based on bioinformatics analysis results, we further validated the role of the JAK2/STAT3 pathway through both *in vitro* and *in vivo* experiments. As displayed in **Figure 8A-B**, we found that the JAK2-STAT3 signaling pathway was significantly activated in NRCM post-LPS stimulation, characterized by a significant increase in the levels of p-STAT3 and p-JAK2 protein expression, whereas Rux treatment significantly inhibited the activation of the JAK2-STAT3 signaling pathway. Subsequently, we detected the JAK2/STAT3 pathway in LPS-induced SCM mice and discovered that both low- and high-dose Rux therapy could reverse the activation of p-JAK2 and p-STAT3 in the mouse heart attacked by LPS (**Figure 8C-D**). Comparable results were observed in the hearts of mice after CLP surgery (**Figure 8E-F**). These results indicated that Rux might exert pharmacological effects by inhibiting the JAK2/STAT3 pathway in the context of SCM. We then examined downstream molecules of the JAK2/STAT3 pathway *in vitro* and *in vivo*, including PSMB8, PSMB9, TAP1, IRF7(**Figure S5**).

The rIL-6 is a commonly used activator of the JAK2-STAT3 pathway^27^, ^43^. As displayed in** Figure 8G-H**, the findings indicated that the concurrent administration of rIL-6 and LPS did not result in additional activation of the JAK2/STAT3 pathway, whereas the utilization of rIL-6 effectively counteracted the inhibitory effects of Rux therapy on the JAK2/STAT3 pathway. Subsequently, we used it to proceed further experiments to investigate whether the regulatory effect of the JAK2/STAT3 pathway on SCM is primarily mediated by mitochondria. The results of **Figure 8I-J** revealed that the stimulation of LPS or the concurrent activation of LPS and r-IL6 had a notable impact on the maintenance of mitochondrial dynamics homeostasis, which was evidenced by an elevation in the levels of p-Drp1 and Fis1 proteins, as well as a reduction in the levels of Mfn2 and Opa1 proteins.

The administration of Rux resulted in the restoration of mitochondrial dynamics, whereas the reactivation of the JAK2-STAT3 pathway using rIL-6 hindered the protective impact of Rux on mitochondrial dynamics. Comparable findings were observed in oxidative stress-related experiments, indicating that the reactivation of the JAK2-STAT3 pathway led to a substantial augmentation in ROS production at both the cellular and mitochondrial levels, as evidenced by the the DCFH-DA assay and MitoSox staining (**Figure 8K-L**). Furthermore, proteins related to mitochondrial-mediated apoptosis were detected. As shown in **Figure 8M-N**, the findings demonstrated that reactivating the JAK2-STAT3 pathway using rIL-6 increased the levels of mitochondrial-mediated apoptotic stress, which Rux had blocked, as evidenced by an increase in the levels of the proteins Bax, Cyto-C, and cleaved-caspase3, as well as a reduction in Bcl2.

### Inhibition of the JAK2-STAT3 pathway is critical for the protective effects of Rux in alleviating cellular senescence and SASP in SCM

**Figure 9:**
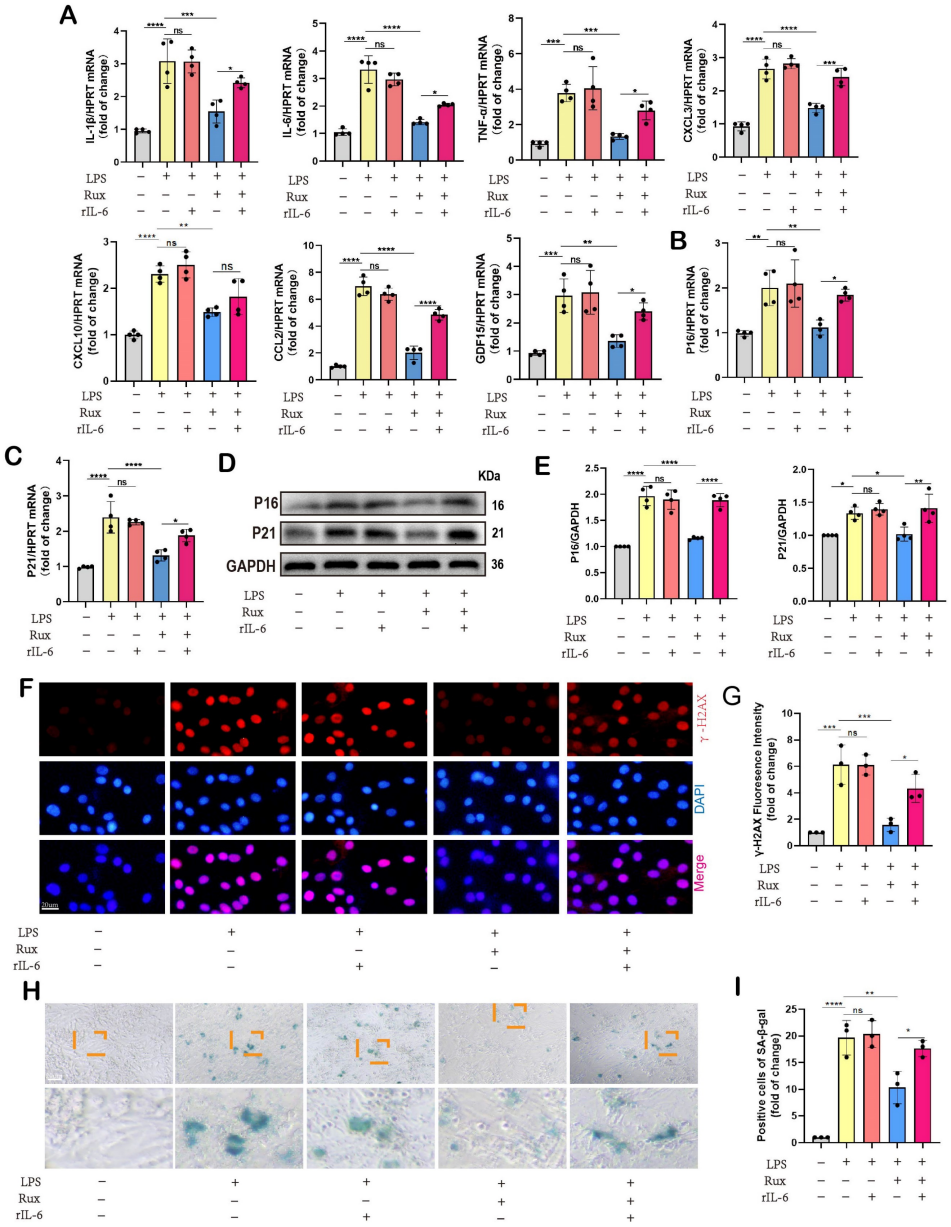
** Inhibition of the JAK2-STAT3 pathway is critical for the protective effects of Rux in alleviating cellular senescence and SASP in SCM.** (A) The mRNA levels of SASP-related genes were detected using qRT-PCR after reactivating the JAK2-STAT3 pathway using r-IL6 in NRCMs, including IL-1β, IL-6, TNF-α, CXCL3, CXCL10, CCL2 and GDF15. N = 4. (B,C) The mRNA levels of P16 and P21 were detected using qRT-PCR after reactivating the JAK2-STAT3 pathway using r-IL6 in NRCMs. N = 4. (D,E) Representative Western blot bands and quantitative analysis of P16 and P21 after reactivating the JAK2-STAT3 pathway using r-IL6 in NRCMs. N = 4. (F,G) Representative fluorescence images and quantitative analysis of γ-H2AX after reactivating the JAK2-STAT3 pathway using r-IL6 in NRCMs. N = 3. (H,I) Representative images and quantitative analysis of SA-β-gal staining after reactivating the JAK2-STAT3 pathway using r-IL6 in NRCMs. Scale bar = 20 μm, N = 3. Data are presented as mean ± SD. * P<0.05, ** P<0.01, *** P<0.001, **** P<0.0001. LPS, Lipopolysaccharide; Rux, ruxolitinib; Con, control; r-IL6, recombinant IL-6; SA-ß-gal, senescence-associated β-galactosidase; NRCMs, neonatal rat cardiomyocytes.

Given the regulatory effect of reactivating the JAK2-STAT3 pathway on mitochondrial dynamics and mitochondrial-mediated apoptosis, we further investigated whether it affected cellular senescence in the context of SCM. Initially, we measured the mRNA levels of SASP-related genes. As displayed in **Figure 9A**, we discovered that following LPS stimulation or the combined use of LPS and r-IL6, SASP factors were significantly induced, including IL-1β, IL-6, TNF-α, CXCL3, CXCL10, CCL2, and GDF15. There was no significant difference between these two groups. Rux therapy reduced SASP factors, however SASP was induced again when rIL-6 was used to reactivate the JAK2-STAT3 pathway. Moreover, Rux therapy reduced the mRNA levels of senescence markers such as P16 and P21 induced by LPS, whereas r-IL6 stimulation restored these levels (**Figure 9B-C**). The protein levels of P16 and P21 exhibited a similar pattern in NRCMs under the same experimental conditions (**Figure 9D-E**). As depicted in** Figure 9F-G**, immunofluorescence results demonstrated that γ-H2AX fluorescence intensity in the nucleus of cardiomyocytes increased significantly following LPS stimulation or the combination of LPS and r-IL6 stimulation, recovered following Rux treatment, and increased again upon co-cultivation of rIL-6. More importantly, as shown in **Figure 9H-I**, SA-β-gal staining further confirmed that reactivating the JAK2-STAT3 pathway significantly increased the positive staining of cardiomyocytes.

**Figure 10:**
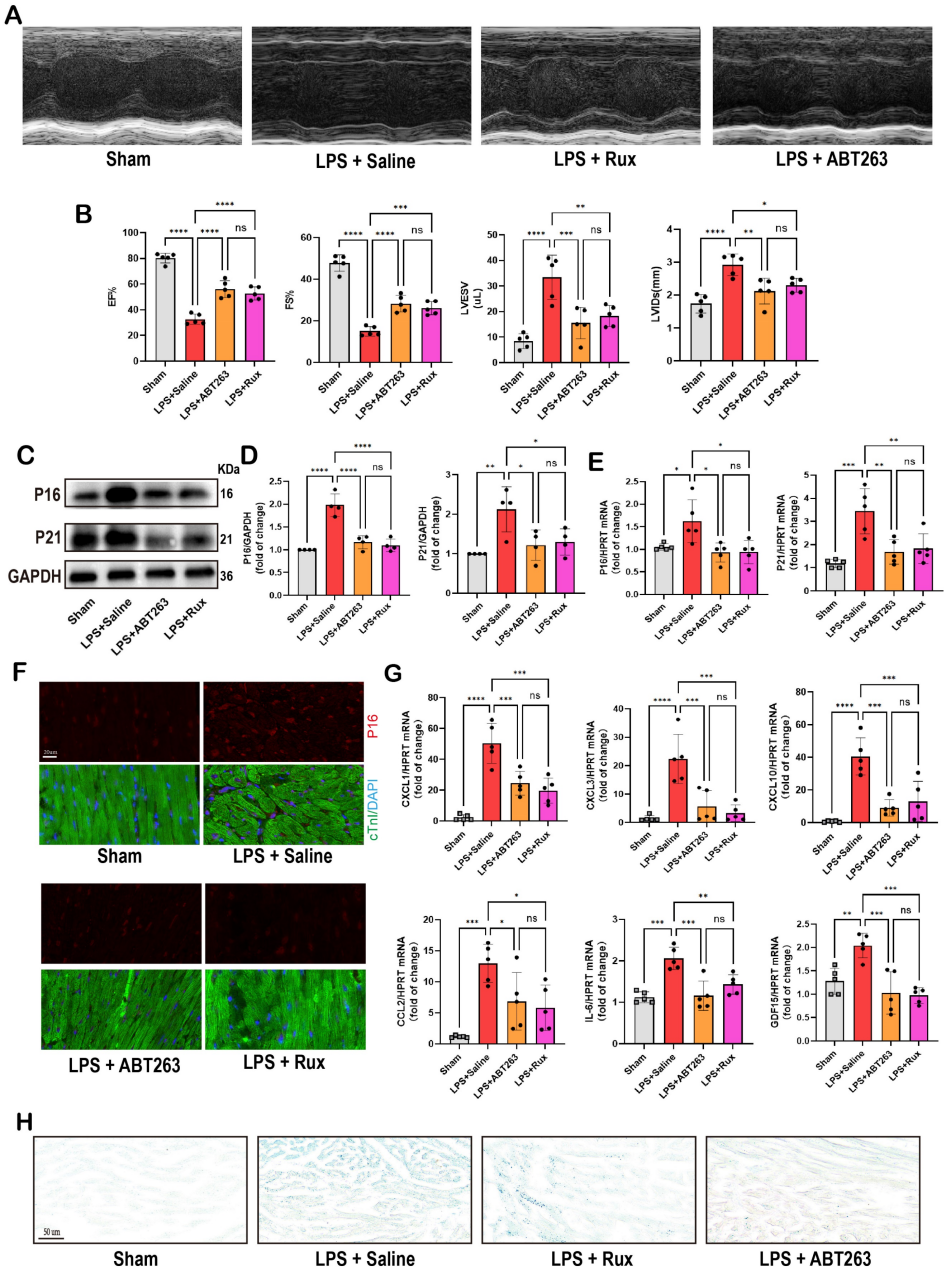
** Low-dose Rux and ABT263 demonstrated comparable efficacy in ameliorating SCM.** (A,B) Cardiac function indices were measured by echocardiography in mice treated with Rux or ABT263 post LPS injection compared with the Sham group. N = 5. (C,D) Representative Western blot bands and quantitative analysis of P16 and P21 in LPS-induced NRCMs treated with ABT263 or Rux. N = 4. (E) The mRNA levels of P16 and P21 were detected using qRT-PCR in NRCMs treated with ABT263 or Rux. N = 5. (F) Representative fluorescence images and quantitative analysis of P16 with cTnI and DAPI in mouse hearts treated with Rux or ABT263 post LPS injection compared with the Sham group. Scale bar = 20 μm, N = 3. (G) The mRNA levels of SASP-related genes were detected using qRT-PCR in mouse hearts treated with Rux or ABT263 post LPS injection compared with the Sham group, including CXCL1, CXCL3, CXCL10, CCL2, IL-6, and GDF15. N = 5. (H) Representative images of SA-β-gal staining in mouse hearts treated with Rux or ABT263 post LPS injection compared with the Sham group. Scale bar = 50 μm. Data are presented as mean ± SD. * P<0.05, ** P<0.01, *** P<0.001, **** P<0.0001. Lipopolysaccharide; Rux, ruxolitinib; SA-ß-gal, senescence-associated β-galactosidase

Overall, the collective findings of our study provide novel insights into the involvement of the JAK2-STAT3 pathway in the modulation of cellular senescence-associated characteristics in the context of SCM. The graphical abstract was displayed in** Figure 10**.

### Low-dose Rux and ABT263 demonstrated comparable efficacy in ameliorating SCM

ABT263, a senolytic pharmacological agent known for selectively eliminating senescent cells and exhibiting benefits in various cardiovascular diseases^44^, ^45^, was further evaluated alongside Rux in the context of SCM. As shown in **Figure 10A-B**, LPS exposure significantly reduced cardiac function in mice. Both ABT263 and low-dose Rux effectively mitigated LPS-induced declines in cardiac function, as evidenced by improvements in EF, FS, LVESV, and LVIDs. Importantly, no significant difference was observed between the effects of ABT263 and Rux on improving heart function. We then evaluated senescence-related markers and SASP. As depicted in **Figure 10C-E**, protein and mRNA levels of P16 and P21 were significantly elevated in the hearts of mice injected with LPS, which were reversed upon treatment with ABT263 and Rux. Immunofluorescence analysis further confirmed consistent expression trends for P16 (**Figure 10F**). Additionally, SASP factors, including CXCL1, CXCL3, CXCL10, CCL2, IL-6, and GDF15, were induced by LPS injection in mouse hearts and attenuated by both ABT263 and Rux treatments (**Figure 10G**). SA-β-gal staining confirmed upregulation of cellular senescence in LPS-stimulated cardiac mice, with subsequent improvement observed following treatment with ABT263 and Rux (**Figure 10H**).

Collectively, these findings indicate that low doses of Rux and ABT263 exhibit comparable efficacy in ameliorating SCM, potentially mediated by their anti-cellular senescence properties.

## Discussion

This study highlighted the potential significance of cellular senescence in the context of SCM, which has not been previously recognized. The study's findings demonstrated the occurrence of cellular senescence in NRCMs, two distinct animal models, and human heart tissues. Significantly, the application of senomorphic therapy using Rux demonstrated a notable reduction in cellular senescence, along with the restoration of mitochondrial function and the decrease of mitochondrial-mediated apoptosis and oxidative stress. Additionally, it is plausible that the JAK2/STAT3 pathway plays a pivotal role in the protective effect of SCM. More interestingly, we found that even low-dose Rux treatment was sufficient to produce similar anti-SCM effects as ABT263. Our study provided innovative insights into strategies for preventing and managing SCM, thereby facilitating future clinical research.

Researchers have been dedicated to identifying “senotherapeutic” approaches that are able to harness senescence for therapeutic purposes, which can be classified into two main categories: senomorphic therapies and senolytic therapies^46^. Senolytic therapy is intended to specifically target and eliminate senescent cells, while senomorphic therapy is a less aggressive approach that aims to prevent harmful cell-extrinsic consequences like SASP without directly causing the death of senescent cells^46^. The widespread adoption of senolytic medication, exemplified by Dasatinib and Quercetin, has sparked a controversy regarding its potential benefits for cardiovascular disease^47^, ^48^. According to a recent study, the elimination of senescent pulmonary endothelial cells through senolytic procedures has been found to potentially exacerbate pulmonary hemodynamics^47^. Researchers have also indicated that senescent cells, such as endothelial cells, adipocytes, and macrophages, were not replenished following their removal and play crucial roles in the aging organism's structure and function^49^, which showed that senolytic interventions should be employed with caution due to their potential adverse effects. Simultaneously, senomorphic agents exhibit considerable potential for future applications. A recent study has revealed that the use of senomorphic drugs to inhibit SASP could reduce the malignant traits of prostate cancer and improve the efficacy of chemotherapy^50^. Regorafenib-based senomorphic treatment effectively reduced senescence and improved porcine pancreatic elastase-induced emphysema in mice^51^. Given the non-renewable nature of cardiomyocytes, delaying senescence using senomorphic therapy could represent a safer tool, which was why we opted for a senomorphic agent to treat SCM instead of senolytic therapy in this study. Recently, researchers identified P16 as a critical regulator of cardiomyocyte senescence and SASP, as evidenced by cardiomyocyte-specific P16 knockout reducing these markers^52^. In this study, we used Adv-mediated gene transfection into the myocardium *in vivo* to induce cellular senescence in mouse hearts (**Figure S6A**). Western blot analysis (**Figure S6B-C**) confirmed successful P16 overexpression in mouse hearts following adenoviral transfection. Treatment with Rux mitigated LPS-induced elevation of P16 levels, which was reversed by Adv-mediated P16 overexpression. Consistent trends were observed in the mRNA levels of P21 (**Figure S6D**). Notably, we observed a decline in cardiac function in mice following LPS administration, which was exacerbated by P16 overexpression (**Figure S6E**). While Rux treatment ameliorated LPS-induced cardiac dysfunction, P16 overexpression partially counteracted this improvement. SA-β-gal staining further confirmed the involvement of cellular senescence in this process (**Figure S6F**). These data suggested a potential role for cellular senescence in SCM mediated by Rux.

Mitochondrial dysfunction is a hallmark of the aging process and cellular senescence, and it has been demonstrated to regulate the SASP^53^, ^54^. Recent studies have demonstrated that senescent cells, previously believed to be resistant to apoptosis^55^, ^56^, displayed signs of mitochondrial apoptotic stress without undergoing cell death 34. This stress, mediated by the involvement of BAX and BAK, resulted in the release of mitochondrial DNA into the cytosol, activating the cGAS-STING pathway. This activation ultimately led to the induction of the SASP^34^. These findings suggested that sublethal mitochondrial apoptotic stress played a significant role in driving the SASP. In the present investigation, a notable increase in the expression levels of proteins associated with mitochondrial-mediated apoptosis was observed in SCM models, namely Cyto-C and Bax, both *in vitro* and *in vivo*. These findings suggested that the occurrence of SCM was accompanied by the induction of mitochondrial apoptotic stress. Most importantly, the administration of Rux demonstrated a mitigating effect on the protein levels of Cyto-C and Bax in NRCMs as well as in mouse models induced by LPS or CLP. These results demonstrated that mitochondrial apoptotic stress might play a crucial role in regulating cellular senescence in the context of SCM, which could be inhibited by Rux threpy.

The impairment of mitochondrial dynamics has a significant role in the facilitation of cellular senescence. A study conducted by researchers has revealed that the deletion of Dnm1p (a homologous gene to Drp1) in fungal models resulted in a reduction in mitochondrial fission, leading to a fungal species characterized by a low growth rate but an extended lifespan^36^. Previous study has also documented that the interaction between Drp1 and filamin, triggered by hypoxia, has the potential to promote mitochondrial hyperfission and myocardial senescence^57^. Enhanced mitochondrial fusion was essential for longevity in the diverse longevity pathways^37^. In the present study, we demonstrated that the administration of Rux therapy had the potential to reinstate mitochondrial dynamics, thereby alleviating the detrimental effects of excessive fission and fusion blockage in SCM, which at least partly explained the anti-senescence effects of Rux in the context of SCM.

MFN2 is a crucial protein in mitochondrial fusion and it participates in the bridging of mitochondria to the ER^58^. Studies have shown that inhibition of MFN2 led to ER stress and MFN2-mediated ER-mitochondrial coupling during ER stress^58^, ^59^. Additionally, the production of ROS could also induce ER stress, which eventually led to cellular senescence^15^. This study revealed that the utilization of Rux had a notable capacity to mitigate ER stress in cases with SCM. This effect might be attributed to enhanced mitochondrial dynamics and diminished stress induced by oxidative stress. In general, the administration of Rux resulted in the restoration of mitochondrial and ER homeostasis, leading to a subsequent reduction in cellular senescence.

Recently, the role of Rux in the context of cardiovascular disorders has emerged. Researchers have identified Rux as a potential cardioprotective CaMKII inhibitor and Rux-treated mice did not show any adverse effects in established cognitive assays with cardioprotective doses of 75 mg/Kg^26^. Research also revealed its cardioprotective effects in coxsackievirus B3-induced acute viral myocarditis^60^. Dong *et al.* identified Rux as a potent and effective drug in the treatment of pulmonary arterial hypertension with the ability of inhibiting the proliferation and migration of rat pulmonary artery smooth muscle cells *in vitro*^61^. Furthermore, it has been documented that the concurrent administration of abatacept and Rux could effectively counteract the progression of fulminant myocarditis caused by immune-checkpoint inhibitors^62^, ^63^. In this study, we found that even low doses of Rux (30 mg/kg) were sufficient to play a protective role in SCM, significantly alleviating cardiac dysfunction. In our CCK-8 assay, we have verified that Rux was not toxic to NRCMs down to 2μM (**Figure S7**). Moreover, Rux significantly inhibited SASP and delayed cellular senescence in both LPS-induced NRCMs and animal models, as well as CLP-induced mouse models. These findings indicated the potential for further clinical research of Rux in the cardiovascular system in the future. Previous studies have suggested that doses of 60 to 90 mg/kg of Rux mice are comparable to human doses ranging from 20 to 25 mg^64^-^66^, yet these assertions lack conclusive pharmacological evidence to substantiate this equivalence. According to the well-established Nair and Jacob scale^67^, the maximum tolerated human dose of 200 mg corresponds to approximately 40 mg/kg in mice^26^, ^68^, ^69^. In our study, we determined that a dose of 30 mg/kg effectively ameliorated septic heart disease in mice, affirming its therapeutic potential. Importantly, this dosage level also aligns with safety thresholds established for human use.

ROS produced by neutrophils have a role in the development of bystander telomere malfunction and senescence in the liver^70^, targeting ROS and ROS-related pathways has been shown to be effective strategies in delaying cellular senescence^71^, ^72^. Furthermore, previous studies have provided evidence indicating that the suppression of mitochondrial antioxidative enzymes, including SOD2 and Txnrd2, contributed to the progression of renal senescence^73^. According to a recent study, SIRT1 has been found to control osteoblast senescence by acetylating SOD2 in the development of osteoporosis resulting from cadmium exposure^74^. These findings indicated that restoring the balance between the antioxidant system and ROS could help mitigate senescence. In this work, data showed that ROS produced at the cellular and mitochondrial levels were inhibited by Rux treatment in LPS-induced NRCMs, accompanied by the up-regulation of SOD2 activity and the inhibition of MDA level. More importantly, we found that both mRNA and protein levels of SIRT3 were suppressed after LPS stimulation in mouse models, but Rux treatment restored their expression levels. SIRT3 has been shown to regulate the deacetylation of SOD2, thereby enhancing its antioxidant activity^35^. In this study, we found that the acetylation level of SOD2 showed an opposite trend to that of SIRT3. Therefore, we believe that Rux treatment may be through up-regulation of SIRT3 expression level, thus deacetylating SOD2, enhancing its antioxidant activity, finally antagonizing ROS production and alleviating oxidative stress in SCM.

The JAK2/STAT3 signaling pathway was reported to regulate oxidative stress, inflammation, and apoptosis in SCM^75^. Although it has not been reported whether the JAK2/STAT3 pathway is associated with cellular senescence in the context of SCM, its role in regulating cellular senescence has been demonstrated in several diseases. Research has found that targeting the JAK2-STAT3 pathway resulted in a reduction of neuronal damage and neuronal senescence^76^. In a mouse model of bleomycin-induced lung fibrosis, it was demonstrated that Nintedanib effectively decreased the quantity of senescence-associated β-galactosidase-positive senescent cells via reducing the phosphorylation of STAT3^77^. The involvement of the JAK2/STAT3 pathway was also observed in the senescence of human glomerular mesangial cells induced by angiotensin II^78^. The current investigation revealed a notable up-regulation of the JAK2/STAT3 pathway in individuals diagnosed with SCM, which was consistent across both NRCMs and mouse models. The administration of Rux had a strong inhibitory effect on the activation of the JAK2/STAT3 pathway. A significant finding was that the reactivation of the JAK2/STAT3 pathway using r-IL6 resulted in the enhancement of SASP, the facilitation of mitochondrial dysregulation, and the promotion of mitochondrial-mediated apoptosis. The findings of this study provided novel evidence on the regulatory function of the JAK2/STAT3 pathway in the process of cellular senescence observed in cases with SCM.

Regarding the limitations of this study, we did not further validate the regulatory effect of reactivating the JAK2/STAT3 pathway on aging phenotype in animal experiments, which will be supplemented in future research. Additionally, it should be noted that our study exclusively utilized male mice; however, it is undeniable that gender may influence the therapeutic efficacy of Rux in treating SCM. Furthermore, inflammation and aging are intricately intertwined, where inflammation not only promotes aging but is also a hallmark of the aging process, often accompanied by chronic inflammation and the SASP 79, 80. The SASP, arising not only from senescent cells but also contributing to their reinforcement through paracrine and autocrine signaling, plays a pivotal role in exacerbating senescence 81. Recent studies underscore the critical role of senescence-induced inflammation as central drivers in diseases such as atherosclerosis. Senescent cells acting as chronic sources of pro-inflammatory cytokines and other factors that destabilize plaques and contribute to various facets of atherosclerosis pathogenesis 82. Recognized as a classic anti-aging agent, ABT263 and other senolytic drugs have demonstrated anti-inflammatory properties 83, suggesting that targeting senescence-induced inflammation could be a promising therapeutic strategy. It is pertinent to note that Rux, evaluated in this study, is known for its potent anti-inflammatory effects. While our investigation partially confirmed the direct involvement of cellular senescence in Rux treatment through cardiac P16 overexpression, inflammation undoubtedly remains a significant participant in the therapeutic process.

## Conclusion

In the present work, we comprehensively demonstrated the occurrence of SCM-induced cardiomyocyte senescence using multiple models. Furthermore, our results suggested that senomorphic therapy with Rux could reduce mitochondrial-mediated apoptosis stress, restore the balance between the antioxidant system and ROS and improve mitochondrial homeostasis *in vivo* and *in vitro*. Mechanistically, the JAK2-STAT3 pathway has been shown to be an important pathway in the regulation of cellular senescence based on Rux therapy in the context of SCM, which might affect cardiomyocyte senescence by regulating SASP, apoptotic stress and mitochondrial dynamics. Overall, this work offers new perspectives and strategies for the treatment of SCM, laying the groundwork for future clinical studies.

## Supplementary Material

Supplementary figures and tables.
