# Supplementary material for: Ruxolitinib-based senomorphic therapy mitigates cardiomyocyte senescence in septic cardiomyopathy by inhibiting the JAK2/STAT3 signaling pathway
Source: Int J Biol Sci. 2024 Aug 12;20(11):4314–40. doi: 10.7150/ijbs.96489 (PMC11379065; doi:10.7150/ijbs.96489)
Supplement: Supplementary file 1 — Supplementary figures and tables. [file ijbsv20p4314s1.pdf]

Ruxolitinib-based senomorphic therapy mitigates cardiomyocyte senescence in septic cardiomyopathy by inhibiting the JAK2/STAT3 signaling pathway

Boshen Yang[1#], Taixi Li[1#], Zhixiang Wang[1#], Yuankang Zhu[2], Kaifan Niu[1], Sien Hu[4], Zhiqi Lin[1], Xinjie Zheng[3*], Xian Jin[1*], Chengxing Shen[1*]

1. Department of Cardiology, Shanghai Sixth People's Hospital Affiliated to Shanghai Jiao Tong University School of Medicine, Shanghai, China.
2. Institute for Developmental and Regenerative Cardiovascular Medicine, Xinhua Hospital, School of Medicine, Shanghai Jiao Tong University, Shanghai, China
3. Department of Respiratory Medicine, The Fourth Affiliated Hospital, Zhejiang University School of Medicine, Yiwu, China
4. Department of Surgery, The Fourth Affiliated Hospital, Zhejiang University School of Medicine, Yiwu, Zhejiang, China

*Correspondence: C.S., shencx@sjtu.edu.cn; X.J., jinxianian@126.com;
X.Z., zhengxinjie@zju.edu.cn

#These authors contribute equally to this work.

Table S1

Briefly, Western blot analyses were performed using commercially available antibodies:

Antibodies		
anti-Bcl2	15071S, CST	1:1000
anti-Bax	5023S, CST	1:1000
anti-Cleaved-caspase 3	9661S, CST	1:1000
Anti-P21	A22460, Abclonal	1:1000
Anti-P16	Sc-1661, Santa	1:100
Anti-P53	A21630, Abclonal	1:1000
Anti-OPA1	A9833, Abclonal	1:1000
Anti-Fis1	AF8268, Beyotime	1:1000
Anti-Mfn2	A19678, Abclonal	1:1000
Anti-Cyto-C	A4912, Abclonal	1:1000
Anti-Sirt3	AF5303, Beyotime	1:1000
Anti-GRP78	AF0171, Beyotime	1:1000
Anti-CHOP	AF6684, Beyotime	1:1000
Anti-ATF4	10835-1-AP, Poteintech	1:1000
Anti-p-JAK2	ET1607-34, HUABIO	1:1000
Anti-JAK2	ab108596.Abcam	1:1000
Anti-p-STAT3	ET1603-40, HUABIO	1:5000
Anti-STAT3	Ab68153, Abcam	1:1000
Anti-γ-H2AX	ET1602-2, HUABIO	1:1000
Anti-GAPDH	HRP-60004, Proteintech	1:10000
Anti-SOD2	ab68155, Abcam	1:1000
Anti-ac-SOD2	ab137037, Abcam	1:1000
Anti-p-Drp1	3455, CST	1:1000
Anti-t-Drp1	8570, CST	1:1000

Table S2. The primer sequences of qRT-PCR.

Primer	Forward	Reverse
Mouse		
P16	CGAACTCTTTCGGTCGTACCC	CGAATCTGAACCGTAGTTGAGC
P21	ATGTCCAATCCTGGTGATGTC	GAAGTCAAAGTTCCACCGTTC
P53	TGGAAGGAAATTTGTATCCCGA	GTGGATGGTGGTATACTCAGAG
Il-6	CCGGAGAGGAGACTTCACAG	TCCACGATTTCCCAGAGAAC
Il-1β	TGCCACCTTTTGACAGTGATG	TGATGTGCTGCTGCGAGATT
TNF-α	AGGGTCTGGGCCATAGAACT	CCACCACGCTCTTCTGTCTAC
Cxcl1	ACCCAAACCGAAGTCATAGCC	TTGTCAGAAGCCAGCGTTCA
Cxcl3	ACCCAGACAGAAGTCATAGCCA	CTTCATCATGGTGAGGGGCT
Cxcl10	CAACTGCATCCATATCGATGAC	GATTCCGGATTCAGACATCTCT
Ccl2	CACTCACCTGCTGCTACTCA	GCTTGGTGACAAAAACTACAGC
Ccl5	GTATTTCTACACCAGCAGCAAG	TCTTGAACCCACTTCTTCTCTG
Gdf15	CCTCCATCTTCTATCTGAGCCTG	CCATGTCGCTTGTGTCCTTTC
Edn3	CCCTGGTGAGAGGATTGTGTC	CCTTGTCCTTGTAAGTGAAGCAC
TGFβ2	CTCGACATGGATCAGTTTATGC	ATAAACCTCCTTGGCGTAGTAC
Sirt3	ATCCCGGACTTCAGATCCCC	CAACATGAAAAAGGGCTTGGG
mt-ND1	CTAATCGCCATAGCCTTCCTAA	GTTGTTAAAGGGCGTATTGGTT
mt-ND2	TTTACCCGCTACTCAACTCTAC	CATCCTATGTGGGCAATTGATG
mt-ND3	CTACTTCCACTACCATGAGCAA	TGTTCATTCATATGCTAGGCCT
mt-ND4	GGATCCACAGCCGTACTATAAT	TGAAGGGGGTAGAGCTAGATTA
mt-ND6	GTTAGTGGGTTTGTTGGTTGTT	CCCAAGTCTCTGGATATTCCTC
mt-Cytb	CCACTCATTCATTGACCTACCT	GCTCCGTTTGCGTGTATATATC

Hprt	GTTGGATACAGGCCAGACTTTGTT	GATTCAACTTGCGCTCATCTTAGGC
18s	ACCGCAGCTAGGAATAATGGA	CAAATGCTTTCGCTCTGGTC
Psmb8	GGACCAGGACTTTACTACGTAG	CAGAATAGTTGTCTCTGTGGGT
Psmb9	CAGGTATATGGAACCATGGGAG	TGCGTCCACATAACCATAAATG
Irf7	AAATAGGGAAGAAGTGAGCCTC	CCCTTGTACATGATGGTCACAT
Tap1	GAATCTTCTCCCTGTTGGTTCC	TGTTCCAGTACAGTAATCCAGC
Primer	Forward	Reverse
Rat		
P16	ATCTCCGAGAGGAAGGCGA	TTGCCCATCATCATCACCTGTA
P21	CAGACCAGCCTAACAGATTTCT	AGACACACTGAATGAAGGCTAA
P53	GCCATCTACAAGAAGTCACAAC	CCAGATACTCAGCATACGGATT
Il-6	ACCAGAGGAAATTTTCAATAGGC	TGATGCACTTGCAGAAAACA
Il-1β	AGGAGAGACAAGCAACGACA	TTGTTTGGGATCCACACTCTCC
TNF-α	AGGGTCTGGGCCATAGAACT	CCACCACGCTCTTCTGTCTAC
Cxcl1	TGCACCCAAACCGAAGTCAT	ACTTGGGGACACCCTTTAGC
Cxcl3	CCCAGACAGAAGTCATAGCCAC	AGGTCCTCCATCACCGTACA
Cxcl10	GTCAGAACAAACTTACCACCAC	AGGAGTTCCACTTTGTACAGTC
Ccl2	CTGTCTCAGCCAGATGCAGTT	TTCTTTGGGACACCTGCTGC
Ccl5	GCTTCAGGTACCATGAAGATCT	GCTGCTGGTGTAAAAATACTCC
Gdf15	TACTCAGTCCAGAGGTGCGA	CTGGTGATGTCCCAGGGC
Edn3	GTTTCACAGGGAAATCGTTTGA	TGAGTGTTTACTTTCGGGAAGA
TGFβ2	GTAAATGCAGCTAAAGTCCTCG	GATGAATCAAAACTCCCTCACG
mt-ND1	CTACATACAACTACGCAAAGGC	GCTTAGAGCTAGTGTAAGGGAG
mt-ND2	TCCTAAACCCAACTATCACCAC	TGATTTTTCGTGTTTGGGTCTG

mt-ND3	CCGAAAAAGCAAACCCATATGA	AGGCGATTTCTAGGTCGAATAG
mt-ND4	CCATCATTCTAGACCCCCTAAC	CGTAGGCAGATTGAGCTAGTTA
mt-ND5	CATGACAAAACCACGATACTCC	AGGATTGTAATGAGTAGGGCTG
mt-ND6	AGTGGATGTATTGGGTGCTTAA	ACCAACTCCATAAAAAGCCCTA
mt-Cytb	ATCTGCTATCCCTTACATTGGG	GTGTTAGGGTTGCTTTGTCTAC
Hprt	GCTGAAGATTTGGAAAAGGTGT	ACAGAGGGCCACAATGTGAT
Psmb8	TATCTGCGGAATGGGGAACG	GCTGCCTGTGGAGAACATCT
Psmb9	GCTGCAAACATAGTGAAGAACA	ATTGCAAAGGGCTGTCGAATTA
Irf7	ACTTAGCCCGGAGCTTGGAT	GTTTGCAACCCAGCATTTCCT
Tap1	GGAGCCCACGATTTCATCTC	ACTCGGGGCTCTCATACAGG

Supplementary Materials

H_2O_2- and D-gal-induced cell models

400μM H_2O_2 was used to stimulate NRCMs for 3 hours to construct a cell model different from that of LPS. The study included a control group of NRCMs, a group of NRCMs treated with 1μM of Rux, a group of H_2O_2-induced NRCMs, and a group of H_2O_2-induced NRCMs treated with 1μM Rux. The Rux therapy began 3 hours after H_2O_2 stimulation until 12 hours. D-galactose (D-gal; #G0750, Sigma-Aldrich, USA) was used at a concentration of 10 g/L. The grouping was similar to that described above. The Rux therapy began 6 hours after D-gal until 24 hours.

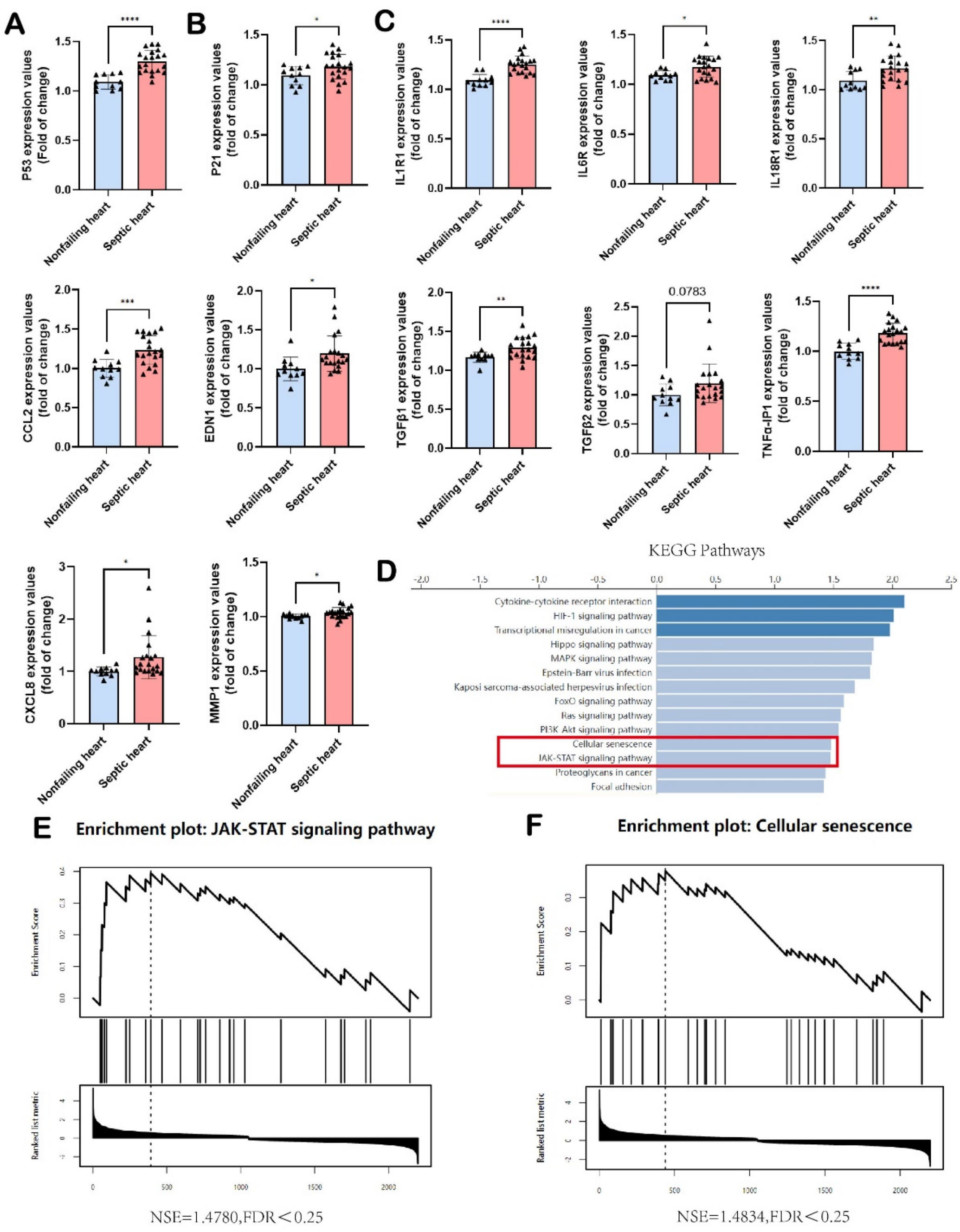

Figure S1 Bioinformatics analysis based on human heart samples obtained from 11 donors whose hearts did not fail and 20 patients with SCM using GSE79962 dataset

(A, B) Relative expression levels of senescence markers, including P21 and P53, between nonfailing hearts and septic hearts. (C) Relative expression levels of SASP-related genes, including IL-1R1, IL-6R, IL-18R1, TNFα-IP1, CCL2, EDN1, TGFβ1, CXCL8, and MMP-1, were compared between nonfailing hearts and septic hearts (D) KEGG analysis revealed that cellular senescence and JAK-STAT signaling pathway were changed between nonfailing hearts and septic hearts (E) GSEA analysis revealed that cellular senescence was induced in septic hearts compared with non-failing

hearts. (F) GSEA analysis revealed that cellular senescence was activated in septic hearts compared with non-failing hearts.

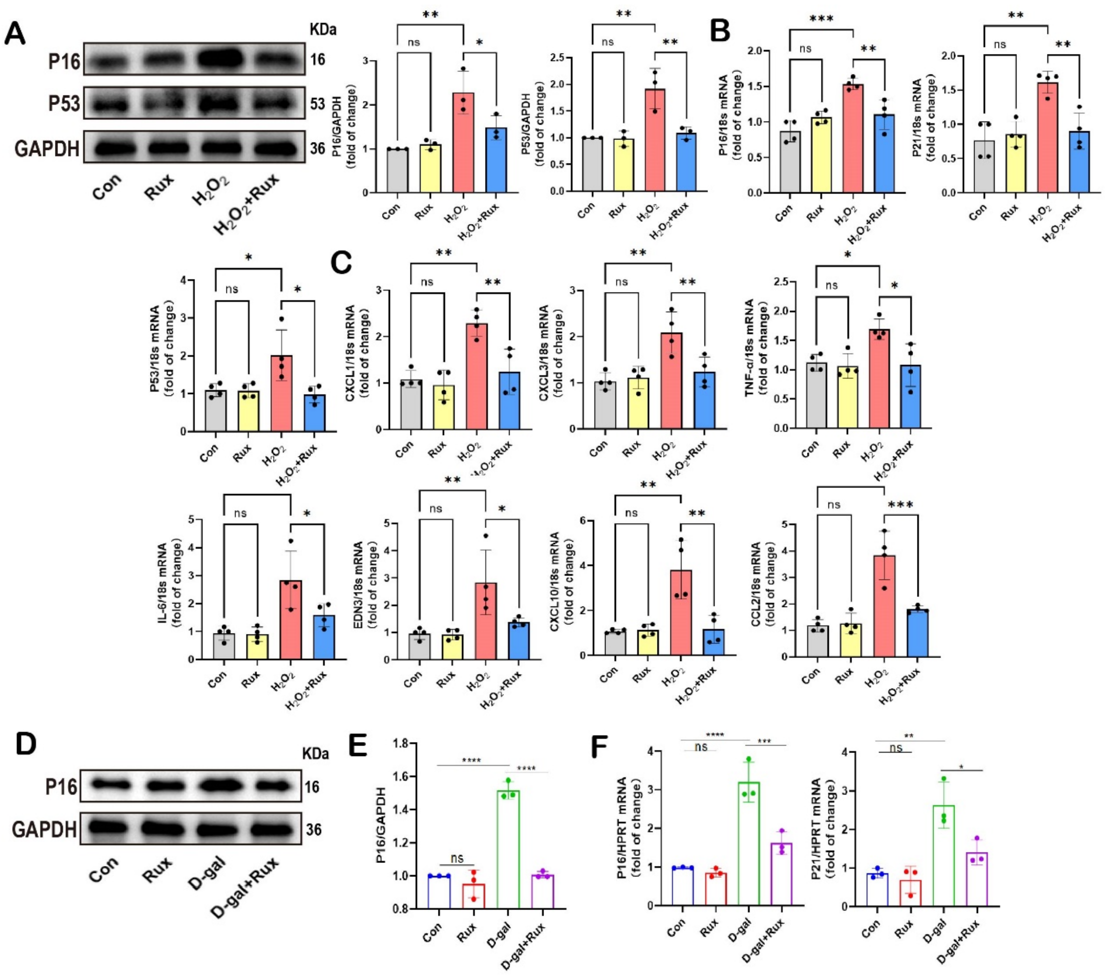

Figure S2 Rux alleviates cellular senescence in H_2O_2- and D-gal induced cell models.

(A)Representative Western blot bands and quantitative analysis of P16 and P53 in NRCMs treated with Rux or not post H2O2 stimulation compared with the control group. N = 3. (B) The mRNA levels of P16, P21 and P53 in NRCMs treated with Rux or not post H2O2 stimulation compared with the control group. N = 4. (C) The mRNA levels of SASP-related genes were detected using qRT-PCR in NRCMs treated with Rux or not post H2O2 stimulation, including IL-6, TNF-α, CXCL1, CXCL3, CXCL10, CCL2, and EDN3. N = 4. (D, E) Representative Western blot bands and quantitative analysis of P16 in NRCMs treated with Rux or not post D-gal stimulation compared with the control group. N = 3. (F) The mRNA levels of P16 and P21 in NRCMs treated with Rux or not post D-gal stimulation compared with the control group. N = 3. Data are presented as mean ± SD. * P<0.05, ** P<0.01, *** P<0.001, **** P<0.0001. Con, control; Rux, ruxolitinib; D-gal, D-galactose.

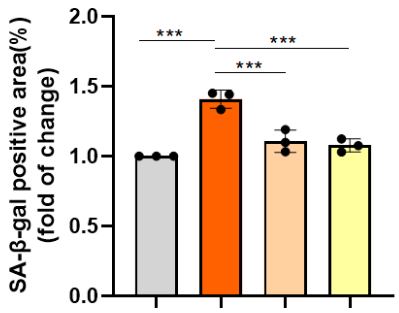

Figure S3

Quantitative analysis of SA-β-gal staining in mouse hearts treated with 30 mg/kg and 75 mg/kg of Rux or saline post CLP-surgery compared with the Sham group. Scale bar = 20 μm. Data are presented as mean ± SD. * P<0.05, ** P<0.01, *** P<0.001, **** P<0. 0001.SA-ß-gal, senescence-associated β-galactosidase.

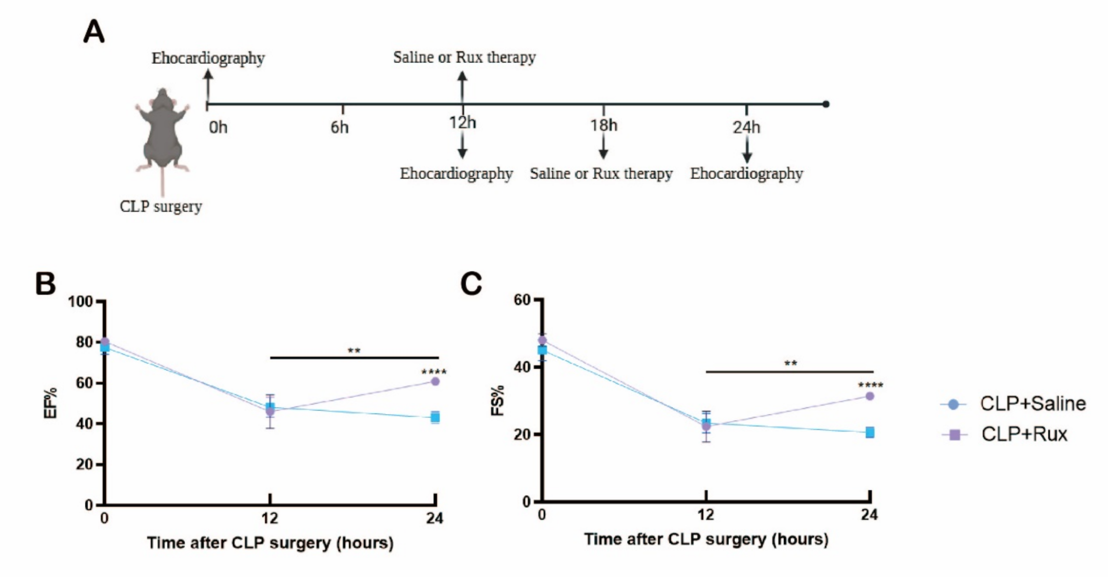

Figure S4 Rux treatment restores cardiac function in the text of SCM.

(A) Schematic diagram of the experiments. (B, C) Cardiac function indices were measured by echocardiography in mice treated with 30 mg/kg Rux or saline post LPS injection compared with the Sham group. N = 5. Data are presented as mean ± SD. * P<0.05, ** P<0.01, *** P<0.001, **** P<0.0001. CLP, cecal ligation and puncture; EF%, ejection fraction; FS%, fractional shortening.

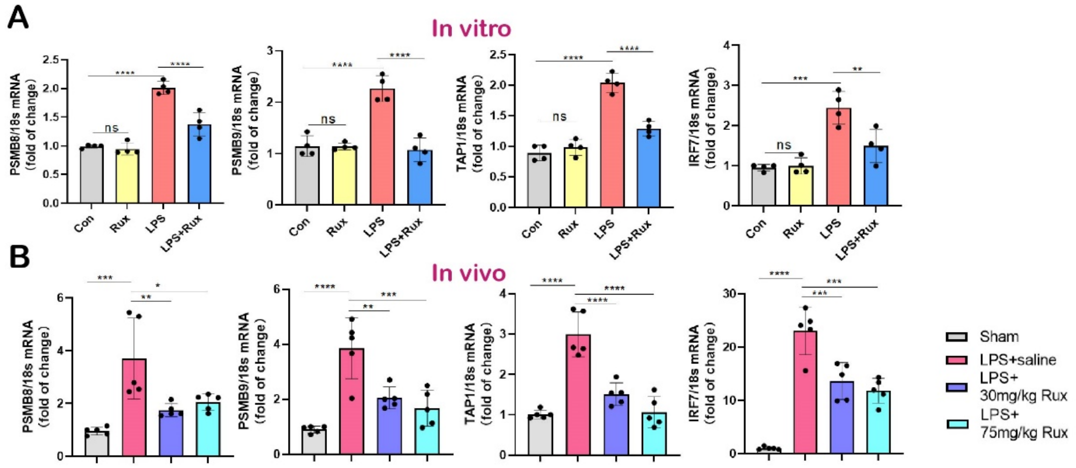

Figure S5 Verification of the downstream genes of the JAK2/STAT3 pathway.

(A)The mRNA levels of PSMB8, PSMB9, TAP1 and IRF7 were detected using qRT-PCR in NRCMs treated with Rux or not post LPS stimulation compared with the control group. N = 4. (B) The mRNA levels of PSMB8, PSMB9, TAP1 and IRF7 were detected using qRT-PCR in mouse hearts treated with 30 mg/kg and 75 mg/kg of Rux or saline post LPS injection compared with the Sham group. N = 5. Data are presented as mean ± SD. * P<0.05, ** P<0.01, *** P<0.001, **** P<0.0001.

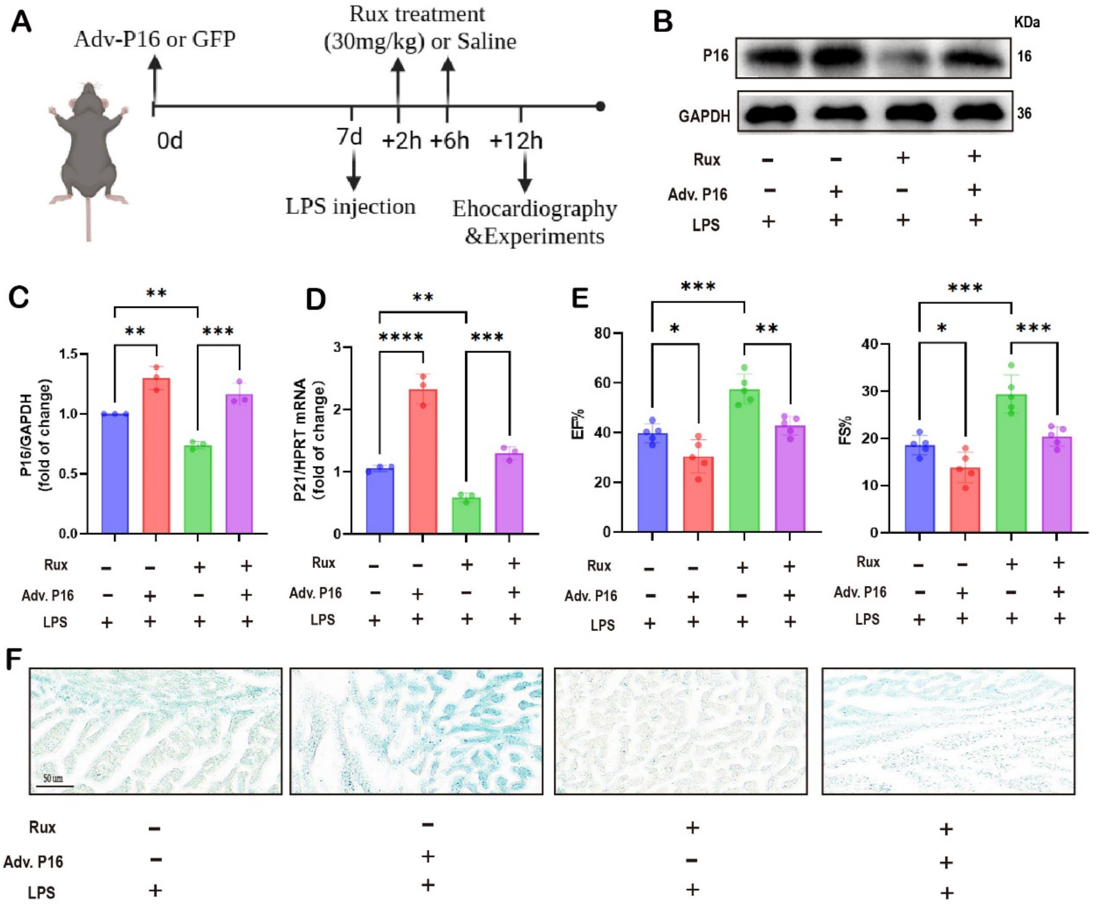

Figure S6 Rux improves cardiac function in septic heart disease partly through cellular senescence. (A) Schematic diagram of the experiments. (B, C) Representative Western blot bands and quantitative analysis of P16 in mouse hearts transfected with adenovirus P16 or GFP treated with Rux or not post LPS injection. N = 3. (D) The mRNA levels of P21 in mouse hearts transfected with adenovirus P16 or GFP treated with Rux or not post LPS injection. N = 3. (E) Cardiac function indices were measured by echocardiography in mouse hearts transfected with adenovirus P16 or GFP treated with Rux or not post LPS injection. N = 5. (F) Representative images of SA-β-gal staining in mouse hearts transfected with adenovirus P16 or GFP treated with Rux or not post LPS injection. Scale bar = 50 μm. Data are presented as mean ± SD. * P<0.05, ** P<0.01, *** P<0.001, **** P<0.0001. Adv, adenovirus; LPS, Lipopolysaccharide; Rux, ruxolitinib.

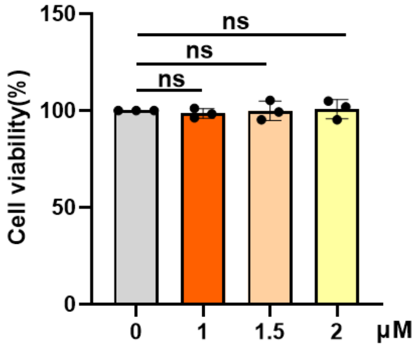

Figure S7 Cell viability assay
